# The role of the microbiome in the neurobiology of social behaviour

**DOI:** 10.1111/brv.12603

**Published:** 2020-05-07

**Authors:** Amar Sarkar, Siobhán Harty, Katerina V.-A. Johnson, Andrew H. Moeller, Rachel N. Carmody, Soili M. Lehto, Susan E. Erdman, Robin I. M. Dunbar, Philip W. J. Burnet

**Affiliations:** 1 Trinity College, Trinity Street, University of Cambridge, Cambridge, CB2 1TQ, U.K.; 2 Leverhulme Centre for Human Evolutionary Studies, Department of Archaeology, Fitzwilliam Street, University of Cambridge, Cambridge, CB2 1QH, U.K.; 3 Institute of Neuroscience, Trinity College Dublin, Dublin 2, Dublin, Ireland; 4 School of Psychology, Trinity College Dublin, Dublin 2, Dublin, Ireland; 5 Department of Experimental Psychology, Radcliffe Observatory Quarter, University of Oxford, Oxford, OX2 6GG, U.K.; 6 Pembroke College, University of Oxford, Oxford, OX1 1DW, U.K.; 7 Department of Psychiatry, Warneford Hospital, University of Oxford, Oxford, OX3 7JX, U.K.; 8 Department of Ecology and Evolutionary Biology, Corson Hall, Tower Road, Cornell University, Ithaca, NY, 14853, U.S.A.; 9 Department of Human Evolutionary Biology, Harvard University, Peabody Museum, 11 Divinity Avenue, Cambridge, Massachusetts, 02138, USA; 10 Psychiatry, University of Helsinki and Helsinki University Hospital, PL 590, FI-00029, Helsinki, Finland; 11 Department of Psychology and Logopedics, Faculty of Medicine, University of Helsinki, P.O. Box 6, FI-00014, Helsinki, Finland; 12 Institute of Clinical Medicine/Psychiatry, University of Eastern Finland, P.O. Box 1627, FI-70211, Kuopio, Finland; 13 Division of Comparative Medicine, Massachusetts Institute of Technology, Building 16-825, 77 Massachusetts Avenue, Cambridge, MA, 02139, U.S.A.

**Keywords:** host–microbe interactions, sociality, autism, emotion, social brain, neurotransmitters, steroids, olfaction, psychobiotics, gene expression

## Abstract

Microbes colonise all multicellular life, and the gut microbiome has been shown to influence a range of host physiological and behavioural phenotypes. One of the most intriguing and least understood of these influences lies in the domain of the microbiome’s interactions with host social behaviour, with new evidence revealing that the gut microbiome makes important contributions to animal sociality. However, little is known about the biological processes through which the microbiome might influence host social behaviour. Here, we synthesise evidence of the gut microbiome’s interactions with various aspects of host sociality, including sociability, social cognition, social stress, and autism. We discuss evidence of microbial associations with the most likely physiological mediators of animal social interaction. These include the structure and function of regions of the ‘social’ brain (the amygdala, the prefrontal cortex, and the hippocampus) and the regulation of ‘social’ signalling molecules (glucocorticoids including corticosterone and cortisol, sex hormones including testosterone, oestrogens, and progestogens, neuropeptide hormones such as oxytocin and arginine vasopressin, and monoamine neurotransmitters such as serotonin and dopamine). We also discuss microbiome-associated host genetic and epigenetic processes relevant to social behaviour. We then review research on microbial interactions with olfaction in insects and mammals, which contribute to social signalling and communication. Following these discussions, we examine evidence of microbial associations with emotion and social behaviour in humans, focussing on psychobiotic studies, microbe–depression correlations, early human development, autism, and issues of statistical power, replication, and causality. We analyse how the putative physiological mediators of the microbiome–sociality connection may be investigated, and discuss issues relating to the interpretation of results. We also suggest that other candidate molecules should be studied, insofar as they exert effects on social behaviour and are known to interact with the microbiome. Finally, we consider different models of the sequence of microbial effects on host physiological development, and how these may contribute to host social behaviour.

## I. INTRODUCTION

All multicellular life hosts microbial life, and the relationships between microorganisms and host lineages appear to be stable over millions of years of host evolution (Moeller *et al*., 2016, 2019; Nishida & Ochman, 2018, 2019). In animals, the majority of these microbes reside in the intestinal tract, where they may number in the trillions. In mammals, microbial colonisation of the host begins during parturition, with the mother’s vaginal and faecal microbes being transmitted to, and subsequently becoming established within, the infant gut (Dominguez-Bello *et al*., 2010; Mueller *et al*., 2015; Ferretti *et al*., 2018; Sprockett, Fukami, & Relman, 2018). The infant microbial community then undergoes substantial reorganisation in response to changes in development, health, and the environment (Koenig *et al*., 2011), but also continues to be shaped by microbial transmission from the mother (Ferretti *et al*., 2018; Moeller *et al*., 2018). The gut microbiome refers to the community of microbes, microbial genes, and the environment they inhabit (Marchesi & Ravel, 2015).

A surge of investigations on the gut microbiome during the past two decades has revealed that these microbes make important contributions to numerous aspects of animal health and physiology across the lifespan (McFall-Ngai *et al*., 2013; Kundu *et al*., 2017; Rook *et al*., 2017). In particular, gut microbes contribute to the regulation of host metabolism, adiposity, and energy balance (Bäckhed *et al*., 2004; Turnbaugh *et al*., 2006; Nicholson *et al*., 2012), as well as appetite and nutrient intake (Perry *et al*., 2016), and the maturation and activity of the immune system (Fung, Olson, & Hsiao, 2017). More recently, gut microbes have been found to influence brain development and function (Diaz Heijtz *et al*., 2011; Braniste *et al*., 2014; Sampson & Mazmanian, 2015; Sharon *et al*., 2016; Vuong *et al*., 2017).

Alongside these effects on the host’s peripheral and central physiology, a growing body of evidence suggests that the microbiome influences host psychological processes such as emotion, learning, and memory (Diaz Heijtz *et al*., 2011; Cryan & Dinan, 2012; Foster & McVey Neufeld, 2013; Dinan *et al*., 2015; Vuong *et al*., 2017; Hoban *et al*., 2018; Sarkar *et al*., 2018). Several investigations in this area are beginning to reveal associations between the microbiome and animal sociality (Hsiao *et al*., 2013; Desbonnet *et al*., 2014; Arentsen *et al*., 2015; Tung *et al*., 2015; Buffington *et al*., 2016; Parashar & Udayabanu, 2016; Stilling *et al*., 2018), and researchers have begun developing hypotheses on the evolutionary and biological mechanisms underpinning microbiome–sociality associations (Montiel-Castro *et al*., 2013; Stilling *et al*., 2014; Archie & Tung, 2015; Münger *et al*., 2018). For an in-depth analysis of these hypotheses in terms of evolutionary theory, see Johnson & Foster (2018).

**Fig. 1.**
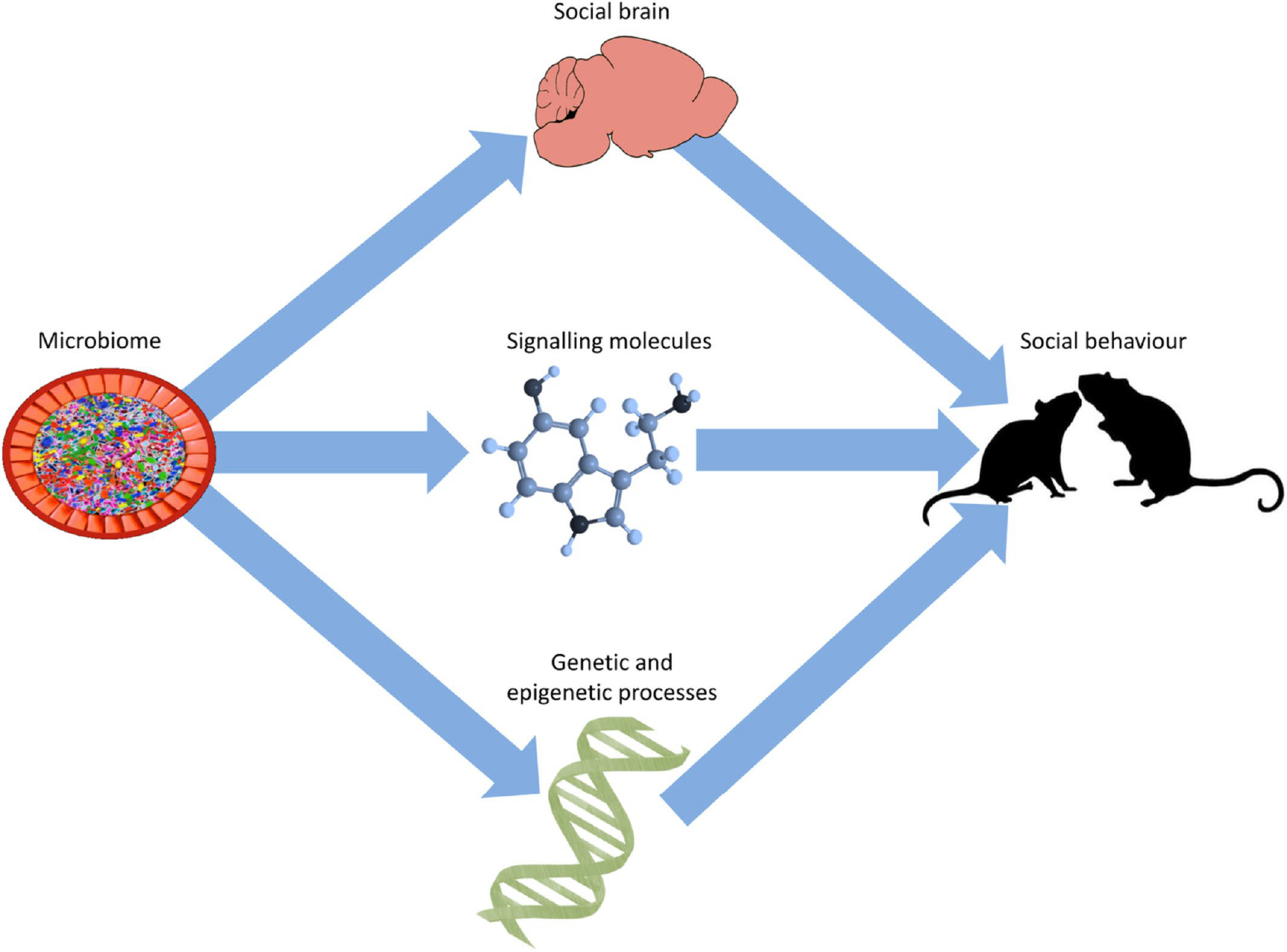
Proposed physiological mediators of the microbiome–sociality relationship. We propose that the microbiome affects host social behaviour *via* regulation of: (*i*) the structure and function of the social brain, (*ii*) signalling molecules known to be involved in social behaviour, and (*iii*) host genetic and epigenetic processes. In addition to the arrows depicted in the diagram, the microbiome’s effects on the structure of the social brain and its signalling molecules may, at least in part, be due to genetic and epigenetic mechanisms.

However, to date there is little evidence that elucidates which causal physiological pathways (at the systems, cellular, and molecular levels) mediate microbial contributions to host social behaviour. Here, we describe three potentially relevant mediators of the link between the microbiome and animal sociality (see Fig. 1). First, the microbiome affects the development and function of brain regions such as the amygdala, hippocampus, and prefrontal cortex (Sudo *et al*., 2004; Hoban *et al*., 2016; Luczynski *et al*., 2016) that are known to contribute to social cognition and social behaviour. Second, the microbiome is capable of generating or regulating the bioavailability of a large number of signalling molecules that influence animal social behaviour, including glucocorticoids, sex hormones, neuropeptides, and monoamines (Sudo *et al*., 2004; Wikoff *et al*., 2009; Markle *et al*., 2013; Poutahidis *et al*., 2013). Finally, the microbiome affects gene expression and epigenetic processes relevant to social behaviour. Although these investigations themselves often do not explicitly link changes in the brain, biochemicals, and gene expression to social behaviour, they do indicate the possible physiological pathways through which the microbiome may influence sociality. Our goal, therefore, is to connect these findings in the context of their relevance to animal social behaviour in order to elucidate some of the physiological mechanisms that may underpin the microbiome–sociality association. Although we focus mainly on the gut microbiome in this review, it should be noted that there are numerous microbiomes distributed across the host body, including the mouth, nose, vagina, and skin, all of which make contributions to host physiology (Dethlefsen, McFall-Ngai, & Relman, 2007; Costello *et al*., 2009; Grice & Segre, 2011).

We first provide a brief overview of the experimental methods used in this field, focussing on pharmacological manipulations, microbial transfers, and germ-free models (i.e. animals that are born and reared in sterile settings, and are therefore devoid of any microorganisms). Then, we adopt a top-down approach, beginning with an overview of experimental investigations of the microbiome–sociality relationship in animals. We synthesise laboratory evidence of the microbiome’s role in the regulation of brain circuitry and signalling molecules implicated in social behaviour. We describe microbial interactions with potential molecular genetic mechanisms underlying animal social behaviour. We consider the contributions of the microbiome to social olfactory signalling in insects and mammals. We also assimilate the emerging research on microbial associations with human emotion and social behaviour, and discuss issues of statistical power and replication. We then focus on the relationship between social behaviour and its underlying physiology and how the microbiome may affect this relationship. Specifically, although the microbiome influences numerous physiological substrates of social behaviour, there is little evidence for microbiome → host physiology → social behaviour pathways. Finally, we describe the importance of attempting to disentangle the order and nature of microbial effects on sociality.

## II. EXPERIMENTAL METHODS IN MICROBIOME–HOST INTERACTION RESEARCH

**Fig. 2.**
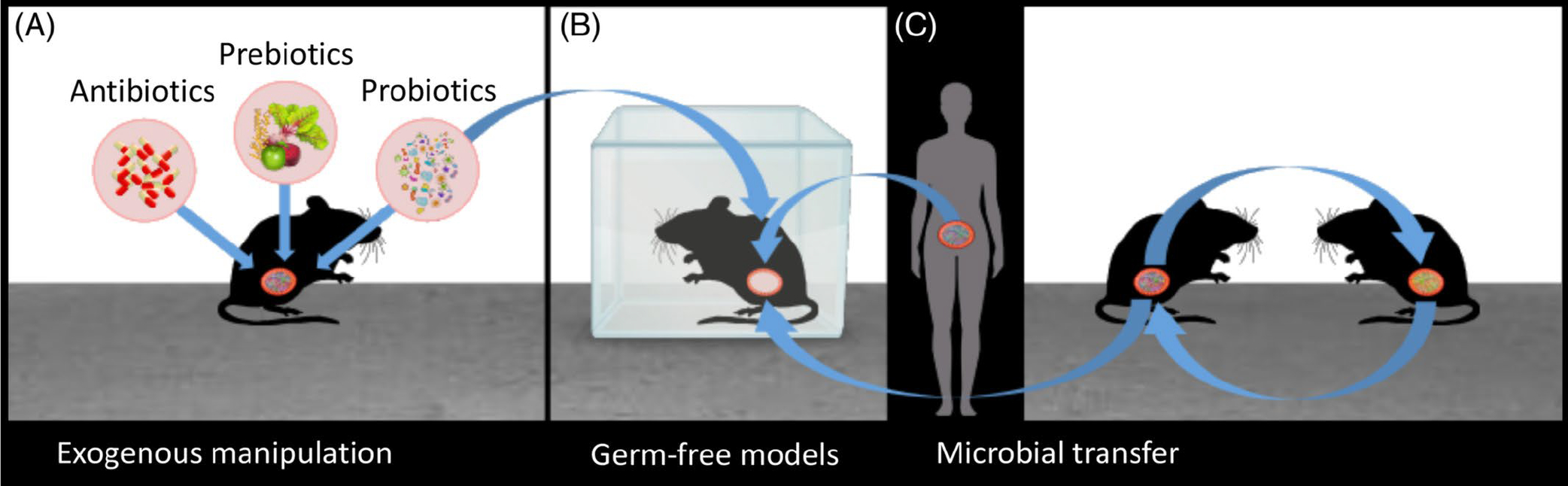
Experimental designs in animal microbiome research.(A)The microbial composition of the gut can be manipulated with antibiotics, probiotics, and prebiotics. (B) Germ-free mice are raised in sterile environments and possess no intrinsic microbes. (C) Microbes can be transferred from one animal to another, either *via* co-housing (i.e. sharing the same physical environment), or by transplantation of faecal matter. The arrows show that germ-free mice can be colonised with specific types of probiotics (monoassociation), with normal or atypical microbiomes from other conspecifics (*via* co-housing or faecal transplants), and with normal or atypical microbiomes from humans in order to evaluate the extent to which the microbiome can recapitulate donor phenotypes in the recipient.

Three of the most common laboratory experimental techniques in investigating host–microbiome interactions are the use of pharmacological or exogenous manipulations (e.g. antibiotics, probiotics, and prebiotics), germ-free models, and microbiome transplants (*via* faecal transfers). As we and others have described these methods elsewhere (Sarkar *et al*., 2018), we cover them only briefly here (see Fig. 2).

### (1) Exogenous manipulations

The microbial content of the gut can be exogenously manipulated using antibiotics, probiotics, prebiotics, and psychobiotics (which are a subset of probiotics and prebiotics).

#### (a) Antibiotics

The effect of antibiotics on gut bacteria depends on the type of antibiotic used and its mode of action. Since antibiotics can and often do ablate non-target microbial populations, they may exert a widespread and significant impact on the host microbiome. Furthermore, not all antibiotic effects necessarily occur *via* modulation of the microbiome (Forsythe, Kunze, & Bienenstock, 2016). For instance, some antibiotic molecules may exert physiological and psychological effects by directly interacting with microglia, enteric neurons, or by modulating enzymatic action (Forsythe *et al*., 2016). Since antibiotic administration studies do not always assess changes in microbial populations directly, it is possible that behvioural outcomes occur *via* antibiotic effects on non-microbial targets. Furthermore, even if researchers do measure changes in the microbiome that covary with a particular behaviour, it does not rule out the possibility that other, non-microbial changes in response to antibiotic exposure may also have contributed to any observed behavioural effects.

#### (b) Probiotics

Probiotics are exogenous live bacteria introduced into the host gut *via* direct ingestion or oral gavage (the latter in the case of animals). Bacteria from the *Bifidobacterium and Lactobacillus* genera are often used as probiotics. Once ingested, these microbes may then have opportunities to colonise the host (perhaps only transiently) and may influence the host’s physiology. However, the incoming probiotics face colonisation resistance in the gut, both from resident microbes (Zmora *et al*., 2018) and the chemical and physical environment of the gut itself (e.g. low pH, rapid effluent flow, secretion of bile, and antimicrobial peptides) (Walter & Ley, 2011). Further research is needed to determine the proportion of ingested probiotics that reach and colonise the gut, dose–response associations, the longevity of probiotic effects, and any possible long-term effects of probiotics on the microbiome (Sarkar *et al*., 2016).

#### (c) Prebiotics

Prebiotics are nutritive resources for microbes, such as indigestible oligosaccharides, that are introduced into the gut to support the growth of beneficial microorganisms. Bacterial fermentation of prebiotics often results in the production of short-chain fatty acids (SCFAs) which can exert a wide range of physiological effects, including on the immune system and metabolism, and the enteric and central nervous systems (Kao, Harty, & Burnet, 2016; Kimura *et al*., 2011; Koh *et al*., 2016). However, some prebiotics are able to exert physiological effects independent of their effects on microbial populations (Forsythe *et al*., 2016). For instance, oligosaccharides may bind directly to the immune system’s pattern-recognition receptors in the lumen or physically prevent these receptors from detecting microbes, with potential anti-inflammatory effects (Bode *et al*., 2004; Eiwegger *et al*., 2010).

#### (d) Psychobiotics

The collection of probiotics and prebiotics that exert psychological effects *via* the microbiome–gut–brain axis are defined as ‘psychobiotics’ (Dinan, Stanton, & Cryan, 2013; Sarkar *et al*., 2016), and researchers may also consider expanding the definition of psychobiotics to include other substances such as antibiotics or dietary components, if their psychological consequences are at least partially mediated by the microbiome (Sarkar *et al*., 2016). In particular, the microbiome is extremely sensitive to the host’s diet (Wu *et al*., 2011; David *et al*., 2014a, 2014b; Carmody *et al*., 2015, 2019; Sonnenburg *et al*., 2016), with diet-induced changes becoming detectable in the microbiome even a day later in some instances (Wu *et al*., 2011; David *et al*., 2014a). As such, we have suggested the possibility that diet could be the strongest source of psychobiotics (Sarkar *et al*., 2018).

### (2) Germ-free models

Germ-free animals are born and raised in microbe-free environments, and are therefore important resources for understanding the influence of microbes on animal physiology. However, it should be noted that germ-free animals differ from conventional animals in terms of both their physiology and social behaviour, and therefore when these animals are colonised by bacteria (e.g. *via* probiotics or microbiome transplants), the results cannot necessarily be extrapolated to animals with normal microbiomes (Hanage, 2014). The most attention is paid to the gut microbiome, which forms the largest and most complex microbial community in the body. The gut microbiome reaches densities in the large intestine that exceed those of other body sites by several orders of magnitude, and the composition of this distal gut community can be inferred non-invasively through DNA sequencing of faecal samples. However, there are also distinct microbial communities associated with other body sites including the skin, mouth, lungs, vagina, and nose, and all of these microbial communities presumably contribute to host health and homeostasis. Germ-free animals lack all of these microbiomes simultaneously and thus it cannot necessarily be deduced that the differences observed in germ-free animals arise solely from the absence of the gut microbiome, given that conventionally colonised animals have numerous other microbiomes which may exert independent and interactive effects on host physiology.

### (3) Microbial transfers

Gut microbes can be transmitted from one animal to another *via* the transfer of faecal matter, which can occur when co-housing animals (a process that is enhanced in coprophagic species), or more directly by transplanting faecal content from one animal to another.

#### (a) Co-housing

Merely housing animals in the same physical environment enables a degree of microbial transfer among individuals. The co-housing approach relies on the environmental and social transmission of microbes among animals (Ridaura *et al*., 2013). In some cases, microbes transferred *via* co-housing can alter phenotypes in recipient mice, including the induction of inflammation (Rehaume *et al*., 2014), as well as affecting other aspects of host physiology. A recent study showed that bacterial transfer *via* co-housing was sufficient to induce immunological changes associated with neurodevelopmental abnormalities in mice (Kim *et al*., 2017). Similarly, microbiome-related social deficits have in some cases been reversed by co-housing experimental and control mice (Buffington *et al*., 2016). These results demonstrate the efficacy of co-housing as a means of microbial transfer in mice, and justify the use of co-housing to at least partially homogenise microbiome composition in mouse experiments (Laukens *et al*., 2016). However, microbial ‘homogenisation’ (or mixing of the microbiomes of co-housed animals) does not consistently occur, and in some cases, microbes may only be transmitted unidirectionally between animals. Specifically, an important study found that mice carrying an ‘obese’ microbiome were sensitive to colonisation by microbes from co-housed mice carrying ‘lean’ microbiomes under specific dietary conditions, but the opposite was not the case (Ridaura *et al*., 2013).

#### (b) Transplantation of faecal microbes

A germ-free mouse can be administered a faecal transplant from a healthy mouse (conventionalisation). Researchers have recently established that these donated faecal microbes can survive in the recipient’s gut for at least 3 months (Li *et al*., 2016). However, microbes from one donor do not always coexist with the recipient’s microbiome to the same extent in different recipients, suggesting that host factors (e.g. host genetics, physiology, or the host’s microbiome itself) can influence the successful establishment of new microbes in the gut.

With appropriate controls, changes in host physiology and social behaviour that follow a faecal transplant can be attributed to the effects of the donor’s microbiome. Germ-free and normally colonised mice can also be colonised with disease-associated microbiomes, either from conspecifics or from humans. In these cases, the microbiome donor has a specific condition (e.g. obesity, anxiety, depression, autism). If microbes are sufficient to induce the physiological or behavioural features of the condition, then faecal transplants to rodents should result in a recapitulation of condition-relevant phenotypes in the recipients, assuming that the faecal microbiome accurately captures the total gut microbiome. While there is evidence that this is the case (Eckburg *et al*., 2005), recent research does suggest that the microbiome associated with the gut mucosa may have limited representation in stool samples (Zmora *et al*., 2018).

Overall, however, faecal transplants allow the inference that the microbiome makes at least some causal contribution to the condition of interest. It is important to keep in mind that these experiments do not necessarily reveal the mechanisms underlying microbial contributions to the condition, or which microbes are involved.

## III. MICROBIAL ASSOCIATIONS WITH SOCIAL STRESS AND SOCIAL BEHAVIOUR

**Fig. 3.**
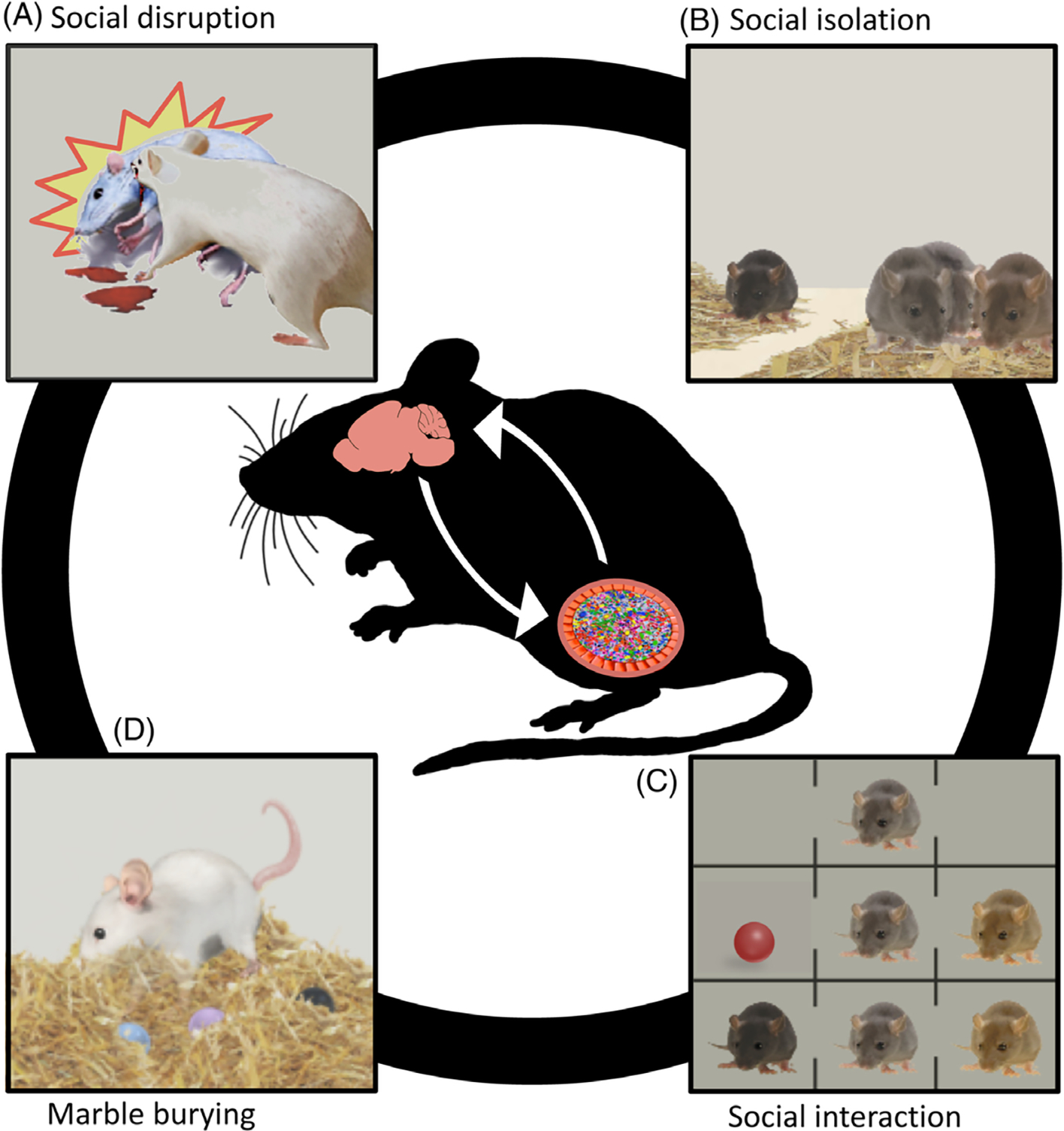
Microbial associations with rodent social behaviour. A range of studies shows that rodent social behaviour is influenced by the microbiome. (A, B) Microbial profiles rapidly shift in response to the social environment, including social defeat stress and social isolation, providing evidence of how the social environment affects the microbiome via the hypothalamic–pituitary–adrenal (HPA) axis. (C) Rodent sociability and social cognition can be influenced by manipulation of the microbiome. In the three-chamber test shown here, the mouse is sequentially exposed to two conditions after habituation (first row). This test measures preferences for social interaction and social novelty. Preference for social interaction is indexed by choosing to interact with a novel conspecific over a novel object (second row). Preference for social novelty is indexed by choosing to interact with a novel mouse over the familiar mouse from the previous phase (third row). Some of the social deficits can be mitigated with probiotic treatment. (D) Disrupting the microbiome can trigger rigid behavioural patterns that are thought to reflect autistic phenotypes such as repetitive behaviour (indexed by marble-burying tendencies), which can be mitigated with probiotic treatment.

We first focus on the association between microbes and host sociality, with an emphasis on social stress and social behaviour (see Fig. 3). We also consider autism, the key features of which include impairments in normal social behaviour. We focus on rodent studies, as most experimental research on microbiome–sociality relationships uses rodents as experimental models (although some studies also examine fish and insects). We illustrate the diversity, potential, and limitations of investigations of the microbiome–sociality relationship. Despite the opportunities that rodent models provide for discovering effects of the microbiome on host behavioural phenotypes, it is important to keep in mind that such findings may not necessarily be extrapolated to humans.

### (1) Social stress in rodents

Stress and negative emotional states significantly alter social interactions, and form a core component of mood disorders and many other psychiatric conditions. In mice, the stress induced by social aggression and subordination to dominant conspecifics triggers changes in the gut microbiome and immune function (Bailey *et al*., 2011; Galley *et al*., 2014; Bharwani *et al*., 2016). Social disruption and social defeat (in which mice are forced to interact with aggressive conspecifics) can reduce gut bacterial diversity (Galley *et al*., 2014; Bharwani *et al*., 2016; Szyszkowicz *et al*., 2017), and can also alter the abundance of specific bacterial taxa. These changes include, for instance, decreases in the relative abundance of the *Bacteroides* and *Lactobacillus* genera (Bailey *et al*., 2011; Galley *et al*., 2014) and increases in the relative abundance of the *Clostridium* genus (Bailey *et al*., 2011). Moreover, some of these changes in bacterial populations occur as early as within 2 hours of exposure to the social stressor (Galley *et al*., 2014), and can last for at least 3 weeks (Szyszkowicz *et al*., 2017), suggesting that microbial responses to the social environment may be both rapid and long-lasting. These microbial changes may occur in parallel with elevations in peripheral proinflammatory cytokines such as interleukin-6 (Bailey *et al*., 2011; Bharwani *et al*., 2016), although this is not always the case (Szyszkowicz *et al*., 2017). Antibiotics have also been observed to attenuate stress-induced proinflammatory immunological activity, further suggesting that gut microbes may mediate the relationship between social stress and inflammation (Bailey *et al*., 2011).

Social stress can also be induced by isolation (Weiss *et al*., 2004). Postweaning separation of rats from conspecifics led to elevations in the Actinobacteria phylum, reductions in the Clostridia class, and an unexpected decrease in hippocampal interleukin-6 (Dunphy-Doherty *et al*., 2018). Insofar as maternal contact during infancy is a crucial form of early social interaction (Feldman, 2017), maternal separation may also be interpreted as a form of social isolation, and is frequently used as a method of inducing stress in young rodents (Meaney *et al.*, 1996; Desbonnet *et al*., 2010). In this regard, maternal separation of rat pups affects gut bacterial composition, reducing the relative abundance of the *Lactobacillus* genus, and elevating concentrations of proinflammatory cytokines (Gareau *et al*., 2007; O’Mahony *et al*., 2009).

While social stress does appear to reliably alter microbial composition, it also seems that different forms of social stress – defeat and aggression (Bailey *et al*., 2011; Galley *et al*., 2014; Bharwani *et al*., 2016), and isolation and separation (Gareau *et al*., 2007; O’Mahony *et al*., 2009; Dunphy-Doherty *et al*., 2018) – trigger different types of changes, with inconsistencies across studies. Aside from the nature of the stressor, other factors that likely contribute to differing effects of social stress on microbial composition include the species, strain, and sex of the rodent, as well as the age at which the stressor is experienced (infancy in the case of maternal separation, adulthood in the case of social defeat and disruption).

Given the bidirectional communication between the gut microbiome and brain, it is possible that the animal’s microbiome can itself affect the stress response. For example, a recent study found that mice which were more resilient to social stress also had a higher prevalence of *Bifidobacterium* in the gut compared to susceptible individuals, suggesting that gut bacteria may buffer against stress (Yang *et al*., 2017). Similarly, social avoidance induced by social stress was found to be most extreme in mice with lower levels of Gram-positive Firmicutes bacteria (*Oscillospira* spp. and *Turicibacter* spp.) and higher levels of Gram-negative Bacteroidetes (*Flavobacterium* spp., *Parapedobacter* spp., and *Porphyromonas* spp.) (Szyszkowicz *et al*., 2017). While these findings are of course correlational, they are at least suggestive of the possibility that certain bacteria may promote psychological resilience against social stress, and as such these potential protective effects warrant further investigation.

### (2) Social behaviour in rodents

A widely used measure of rodent social behaviour is the three-chamber test (see Fig. 3), which provides an index of rodent sociability and social cognition (Nadler *et al*., 2004; Moy *et al*., 2007; Silverman *et al*., 2010; Yang, Silverman, & Crawley, 2011). The task involves two steps, following an initial habituation phase. First, the rodent is placed in the middle of three interconnected chambers. One of the adjacent chambers contains an unfamiliar conspecific, while the other contains a novel object (alternatively, this chamber may be empty). Normal rodent sociability is indexed by greater behavioural preference for the conspecific. The second step also involves three interconnected chambers. In this case, the adjacent chambers contain a familiar rodent (from the first step) and an unfamiliar rodent. Typical social cognition is indexed by greater behavioural preference for the unfamiliar conspecific. Disturbances in sociability and social cognition are reflected in reduced interest in the conspecific (step 1) and the unfamiliar conspecific (step 2), respectively.

This three-chamber test is frequently used to assess social behaviour in germ-free rodents in microbiome experiments. For instance, unlike their normally colonised counterparts, germ-free mice exhibit social impairments in the three-chamber test. In particular, they do not show the normal preference for interacting with other rodents (impaired sociability), nor a preference for interacting with an unfamiliar mouse over a familiar one (impaired social cognition) (Desbonnet *et al*., 2014; Buffington et al., 2016; Stilling *et al*., 2018). Microbial reconstitution attenuated the impairments in sociability, but did not ameliorate social cognition (Desbonnet *et al*., 2014; Stilling *et al*., 2018), suggesting that some – but not all – of the social deficits may be reversible. However, because both sociability and social cognition were each only tested once in these studies, it may also be the case that changes in social cognition occur more slowly than changes in sociability, and may therefore be apparent only in further testing sessions. Similar to germ-free mice, germ-free rats also show impairments in sociability in the early stages of a social interaction task (Crumeyrolle-Arias *et al*., 2014). Overall, these results provide causal evidence that some aspects of normal host sociality may require the presence of a microbiome.

However, there is one intriguing report that germ-free status *increased* sociability in mice, as observed in the three-chamber test (Arentsen *et al*., 2015). The mice used in this study were older than those used in some of the research that found that germ-free status decreased sociability (Desbonnet *et al*., 2014; Stilling *et al*., 2018), and this may account for the divergent effects of germ-free status on sociability. The hypothesis that an animal’s age may affect how its microbiome influences its social behaviour could be tested by systematically examining social interactions in germ-free mice of different ages.

### (3) The rodent gut microbiome and autism

#### (a) Associations with the gut microbiome in rodent models of autism

The microbiome has been implicated in autism, which is a complex condition defined by deficits in social communication and interaction, as well as rigid and repetitive behavioural patterns (Baron-Cohen & Belmonte, 2005; Happé, Ronald, & Plomin, 2006). Autism is often also associated with gastrointestinal and immunological disturbances (Horvath & Perman, 2002; Ashwood *et al*., 2011; Patterson, 2011; Onore, Careaga, & Ashwood, 2012; McElhanon *et al*., 2014). Gastrointestinal and immunological processes are in turn associated with the microbiome, and as such, the nature of microbial involvement in the multidirectional relationships between the gastrointestinal system and the immune system in autism are unclear, and are an important area of investigation (Azhari, Azizan, & Esposito, 2019).

A rapidly growing body of research is beginning to suggest ways in which the microbiome may be functionally involved in autism (Vuong *et al*., 2017; Vuong & Hsiao, 2017), raising the possibility that the microbiome may contribute to its aetiology. For instance, research in rodents shows that maternal experiences can disturb microbial composition in the offspring. These maternal experiences include exposure to antibiotics (Degroote *et al*., 2016), acute systemic inflammation (i.e. maternal immune activation; Hsiao *et al*., 2013; Kim *et al*., 2017; Lammert *et al*., 2018; Morais *et al.*, 2018), or consumption of high-fat diets (Buffington *et al*., 2016), all of which alter the offspring’s microbiome. Crucially, these microbial perturbations are associated with behavioural profiles consistent with autistic traits, including reduced sociability and repetitive behaviour [assessed, for example, by excessive burying of marbles (Thomas *et al*., 2009; Malkova *et al*., 2012)].

In particular, a recent study (Buffington *et al*., 2016) found that pregnant mice that consumed high-fat diets gave birth to offspring that showed autistic-like phenotypes. When healthy mice engaged in regular social interactions, long-term potentiation occurred in the ventral tegmental area. In comparison, the autistic-type mice showed comparatively lower levels of long-term potentiation in the ventral tegmental area after social interactions, and also had fewer oxytocin-expressing neurons. The causal role of the microbiome was revealed using faecal transplants to transfer microbes from the autistic-type mice to the control mice: the recipients developed social behavioural deficits and showed impaired long-term potentiation in the ventral tegmental area, as well as reductions in oxytocin-expressing neurons. This suggests that the microbiome is able to induce autistic-like phenotypes in neurotypical recipients.

Perhaps most striking, however, is the finding that probiotic treatment with *Lactobacillus reuteri* and *Bacteroides fragilis* ameliorated some of the autistic-like phenotypes in mice (Hsiao *et al*., 2013; Buffington *et al*., 2016). While of course still very far from clinical application to humans, such rodent findings nonetheless provide early evidence that some of the behavioural features of complex neurodevelopmental conditions may be at least partially reversible in some cases through exogenous manipulation of the gut microbiome.

The specific pathways through which the microbiome may contribute to autistic-like behaviours are still largely unknown and in need of rigorous mechanistic elucidation. However, recent efforts using the maternal immune activation model of autism in rodents have begun to uncover microbiome–immune associations that affect the likelihood of developing autistic phenotypes in response to inflammation during pregnancy. In mice, elevations in maternal concentrations of the proinflammatory cytokine interleukin-17a produced by T helper 17 (T_H_17) cells may mediate the relationship between maternal infection during pregnancy and infant autistic phenotypes (Choi *et al*., 2016). Signalling by T_H_17 cells and interleukin-17a during pregnancy appears to rely on the presence of segmented filamentous bacteria in the maternal gut (Kim *et al*., 2017). Maternal immune activation in the absence of T_H_17-promoting segmented filamentous bacteria in the gut does not produce autistic-type offspring (Kim *et al*., 2017). However, when mice that were lacking segmented filamentous bacteria were then exposed to these bacteria, either directly or through interactions with other mice carrying these bacteria, maternal immune activation did trigger autistic phenotypes in the offspring *via* elevations of interleukin-17a (Kim *et al*., 2017; Lammert *et al*., 2018). These results suggest that maternal microbes may be acting as environmental risk factors for autism.

The genetic background of the host may also moderate the effects of environmental risk factors on the development of autism. Host genes are known to exert some influence on the composition of the microbiome (Goodrich *et al*., 2014), and therefore host genetic factors may also influence the microbiome–autism association. For instance, in a comparison of the effects of maternal immune activation on autistic traits between C57BL/6J mice and NIH Swiss mice, the latter were found to bury significantly more marbles than the former, although sociability was similarly impaired in both strains following the intervention (Morais *et al*., 2018).

Genetic research on autism in humans has implicated *SHANK* family genes in the aetiology of autism (Jiang & Ehlers, 2013). *SHANK* genes (*SHANK1*, *SHANK2*, and *SHANK3*) encode synaptic folding proteins, and genetic manipulations that alter the expression of these proteins have been used to model the effects of genetic risk factors of autism (Jiang & Ehlers, 2013). A recent gene-knockout study found that mice lacking *Shank3* displayed autistic-like phenotypes (e.g. impaired sociability and repetitive behaviours) alongside several changes in gut bacterial composition and reductions in the expression of *γ*-aminobutyric acid (GABA) receptors in the hippocampus and prefrontal cortex (Tabouy *et al*., 2018). Crucially, treatment with the probiotic *Lactobacillus reuteri* attenuated the behavioural deficits and also increased expression of GABA receptors in the affected brain regions (Tabouy *et al*., 2018). Therefore, *Lactobacillus reuteri* appears to diminish autism-related phenotypes in two distinct murine models of autism (Buffington *et al*., 2016; Tabouy *et al.*, 2018).

Indeed, researchers have now followed this lead to show explicitly that *Lactobacillus reuteri* appears to be effective in treating murine autism symptoms with diverse aetiologies (Sgritta *et al*., 2019). These include environmental models (maternal exposure to valproic acid), genetic models (*Shank3* knockout), and idiopathic models (BTBR mice show autistic traits but there are no known genetic or environmental sources, and as such these mice are considered to represent idiopathic autism). In all cases, treatment with *Lactobacillus reuteri* ameliorated the social deficits associated with these conditions (i.e. increased time in social interactions, increased sociability, and increased preference for social novelty compared to untreated mice). Vagotomy (i.e. surgical removal of the vagus nerve) abolished probiotic benefits, suggesting that the behavioural benefits of *Lactobacillus reuteri* are mediated by the vagus nerve. Moreover, monoassociation of germ-free mice with *Lactobacillus reuteri* also rescued social functioning (Sgritta *et al*., 2019). These results suggest that this probiotic can exert its effects independent of other microbes and that it can rescue social impairments in diverse mouse models of autism.

Another recent study sought to examine the effects of transplanting gut microbes from autistic humans to mice (Sharon *et al*., 2019). Germ-free mice were colonised using faecal transplants from neurotypical or autistic donors, with the autistic donors for this study comprising 11 individuals with mild, moderate, and severe autism. This initial generation of colonised mice was then used to breed a second generation. In particular, each member of the second generation was bred from parents which had received microbiome transplants from the same human donor. The gnotobiotic conditions meant that vertical transmission of microbes could only include microbial populations derived from human donors, as those were the only microbes that had colonised the parents. This allowed for an examination of the causal contributions of the microbiome to autism in the offspring.

The researchers did not observe any differences between the mice carrying microbiomes derived from autistic donors compared to mice carrying microbiomes derived from neurotypical donors in the three-chamber test. However, the experimental mice did show reduced social engagement with conspecifics in a separate test investigating direct social interactions, and also buried significantly more marbles compared to the control group (although in this latter case, the effect was only apparent when excluding mice whose microbiomes were derived from donors diagnosed with mild autism). However, subsequent work by independent researchers suggested that there may have been software-associated technical issues in the original analysis that led to errors in the estimates of statistical significance in the results. In particular, researchers have suggested that the mouse data may have been analysed as if each mouse received microbes from independent donors, whereas in fact all of the mice were colonised by microbes from one of 11 donors (meaning that multiple mice received transfers from the same donor). It appears that correcting for this issue leads to a loss of statistical significance in the case of social interaction, although the differences in marble burying remained statistically significant. Overall, therefore, it will be crucial to replicate these results using a wider pool of autistic and neurotypical donors.

#### (b) Drawbacks to rodent models of autism and potential alternatives

In general, there is much debate over the utility of rodent models of autism, and there is as yet no universally accepted rodent model that is considered equivalent to the behavioural impairments associated with autism in humans. While of course atypical sociality and repetitive behaviour in mice provide an attractive resemblance to human autism, it is far from clear whether these behavioural impairments in rodent models are effective at genuinely capturing the vastly more complex phenotypes of human autism. Thus, while the results of microbiome–sociality studies in rodents are certainly provocative and conceptually interesting, the distance between rodent ‘autism’ and human autism poses a significant translational barrier. Initiating human clinical trials on the basis of only rodent results would be extremely resource intensive and may not yield any meaningful results, and, moreover, may unnecessarily subject young participants to discomfort or distress associated with the testing procedures. One solution that we have suggested previously is the use of primate models after preclinical rodent results have been established (Sarkar *et al*., 2018). In this regard, researchers have recently developed a macaque (*Macaca fascicularis*) model of autism with *SHANK3* mutations using the CRISPR–Cas9 (clustered regularly interspaced short palindromic repeats–CRISPR-associated protein 9) gene-editing system (Zhou *et al*., 2019). Crucially, alongside disturbances in neurocircuitry, the macaques showed social impairments and repetitive behaviour reminiscent of the hallmark features of autism. As such, it may be worthwhile to consider, where feasible, how microbial interventions affect autism-relevant phenotypes in macaques prior to initiating human investigations. Though such primate studies would themselves be highly resource intensive, in the long run they would likely be more efficient if conducted as follow-ups to rodent studies and prior to human studies.

## IV. MICROBIAL INFLUENCES ON THE SOCIAL BRAIN

**Fig. 4.**
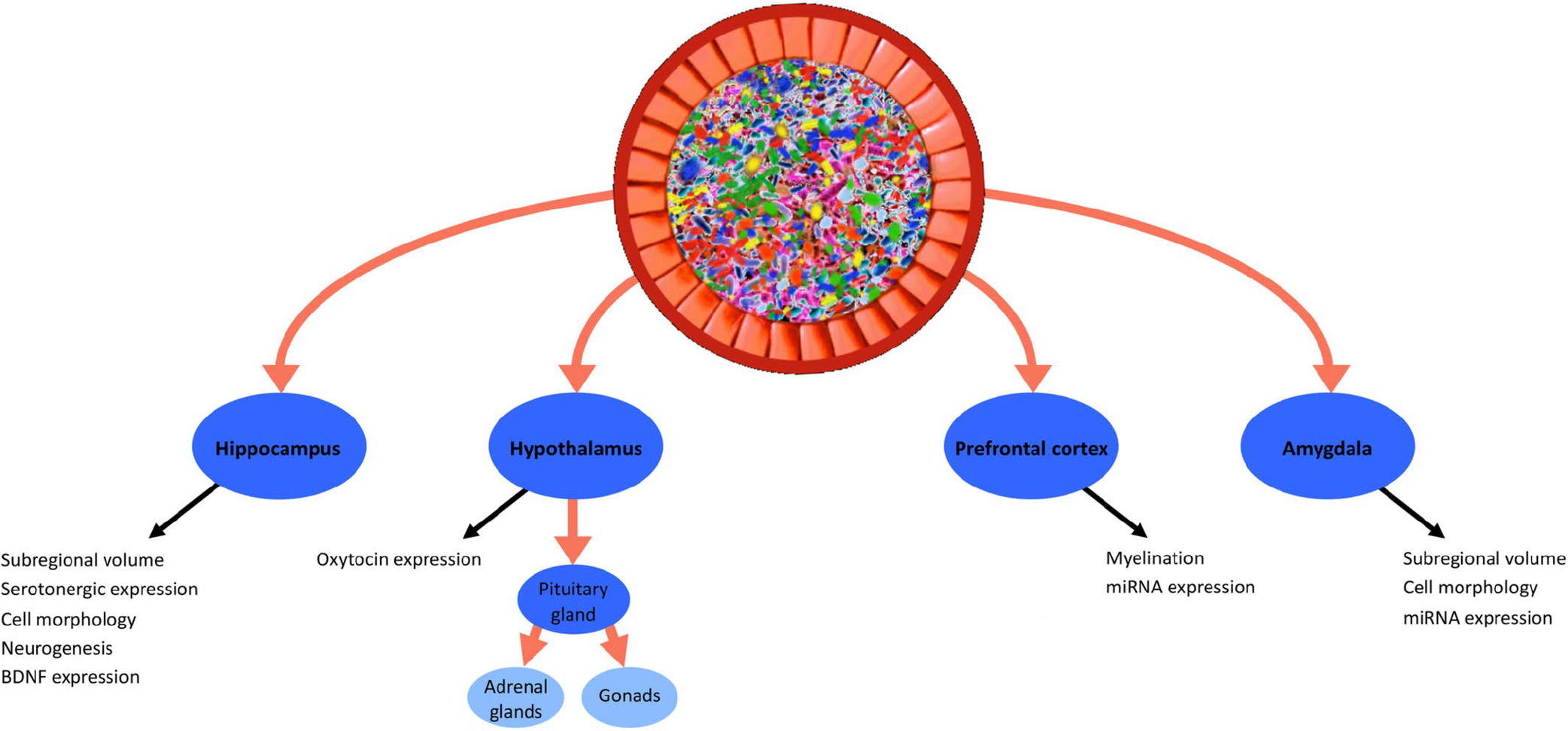
Microbial influences on the social brain. The gut microbiome exerts a range of effects on the development and function of the social brain. These include hippocampal neurogenesis, volumetric and morphological alterations in the amygdala and hippocampus, prefrontal myelination, and hypothalamic oxytocin expression, as well as the development of both the hypothalamic–pituitary–adrenal axis and hypothalamic–pituitary–gonadal axis. BDNF, brain-derived neurotrophic factor; miRNA, microRNA.

Gut microbes make important contributions to brain development and function (see Fig. 4), including the amygdala and the prefrontal cortex, both of which are crucial nodes in the network comprising the ‘social’ brain. In addition, the microbiome has been found to affect the hippocampus, which also plays a role in social cognition. The microbiome also influences the hypothalamus, which regulates a range of signalling molecules that exert well-known social effects.

### (1) Amygdala

The amygdala is a subcortical brain structure that plays an important role in processing social-affective information (Phelps & LeDoux, 2005), and mediates the experience of stress, fear, and anxiety (Roozendaal, McEwen, & Chattarji, 2009). On the other hand, reduced amygdalar activity during social perception tasks is hypothesised to be associated with autism and autistic-type traits in humans (Baron-Cohen et al., 1999, 2000). More recently, researchers have also observed ‘simulation’ neurons in the primate amygdala (Grabenhorst *et al*., 2019). Specifically, these neurons appear to facilitate the simulation of the mental states of a monkey’s social partners (Grabenhorst *et al*., 2019).

Several studies have revealed that the microbiome exerts effects on the structure and function of the amygdala (Cowan *et al*., 2018). For example, in germ-free mice, the lateral amygdala, the basolateral amygdala, and the central nucleus of the amygdala have a greater volume compared to normally colonised controls (Luczynski *et al*., 2016). Dendritic hypertrophy has also been observed in the basolateral amygdala of germ-free mice. In particular, the dendrites of aspiny interneurons of germ-free mice were both longer and had a greater number of branch points compared to normally colonised controls (Luczynski *et al*., 2016). The dendrites of pyramidal neurons in the basolateral amygdala of germ-free mice were also longer, with increased density of thin spines, stubby spines, and mushroom spines (Luczynski *et al*., 2016). In mice, ingestion of the probiotic *Lactobacillus rhamnosus* lowers amygdalar expression of GABA_Aα2_ messenger ribonucleic acid (mRNA) (Bravo *et al*., 2011). The microbiome also affects other aspects of gene expression in the murine amygdala, which we discuss later (see Section VI).

There is also some evidence of a possible link between the gut microbiome and the human amygdala, although it is far less robust than findings in rodents. In particular, higher levels of intrinsic *Prevotella* spp. in healthy volunteers were associated with greater white matter connectivity between the amygdala and the caudate (Tillisch *et al*., 2017). Higher levels of Actinobacteria were also found to be positively correlated with fractional anisotropy of the amygdala (with higher fractional anisotropy in turn predicting better microstructural organisation) (Fernandez-Real *et al*., 2015). Researchers have also found preliminary evidence of an association between microbial diversity and the functional connectivity between the amygdala and the thalamus (Gao *et al*., 2019). However, it is important to note that since these are correlational studies, it may be that the relationship between the microbiome and the amygdala is mediated by stress, since stress can affect both the amygdala and microbiome composition.

Two reward-related networks, the amygdala–nucleus accumbens circuit and the amygdala–anterior insula circuit, have also recently been shown to be associated with microbially generated indole metabolites in humans (Osadchiy *et al*., 2018). In particular, the concentrations of different indole metabolites (indole, indoleacetic acid, and skatole) obtained from faecal samples were positively correlated with both anatomical and functional connectivity in the amygdala (Osadchiy *et al*., 2018). Moreover, consumption of probiotics (relative to controls) has been found to reduce activity in a brain network implicated in processing emotional information, including the amygdala, in a group of healthy female volunteers (Tillisch *et al*., 2013). Notably, studies have also failed to detect correlations between bacterial profiles and amygdalar volume in comparisons of healthy individuals and those diagnosed with irritable bowel syndrome (Labus *et al*., 2017; Tillisch *et al*., 2017). As such, the strength of the association between the microbiome and the amygdala remains to be clarified. More generally, though intriguing, these reports will need to be followed up with larger investigations in order to determine the nature of the microbiome–amygdala relationship with greater specificity and to test replicability.

### (2) Prefrontal cortex

The prefrontal cortex is involved in high-level cognition and executive functions (Miller & Cohen, 2001), and also makes key contributions to social cognition, including impression formation (Mitchell, Macrae, & Banaji, 2005), learning social value (Behrens *et al*., 2008), and social and moral reasoning (Anderson *et al*., 1999). Furthermore, in humans, the prefrontal cortex is associated with social network size both volumetrically (Lewis *et al*., 2011) and functionally (Noonan *et al*., 2014, 2018), relationships that appear to be evident in other primates as well (Sallet *et al*., 2011).

Germ-free status in mice triggers morphological abnormalities in the prefrontal cortex, particularly enhanced thickness of the myelin sheath and an upregulation of genes associated with myelination and myelin plasticity (Hoban *et al.*, 2016). Microbial transfers from stressed mice have also been found to trigger prefrontal demyelination and social avoidance in healthy recipients (Gacias *et al*., 2016), suggesting that the effects of stress on the brain may be at least partially mediated by the gut microbiome. Furthermore, given that social isolation in mice impairs adult prefrontal myelination (Liu *et al*., 2012) and that social isolation itself affects the microbiome (Gacias *et al*., 2016; Hoban *et al*., 2016), it is reasonable to hypothesise that some of the effects of social isolation on myelination of the prefrontal cortex may be microbially mediated. There is also evidence that the prefrontal cortex is sensitive to probiotics. In particular, mice that were treated with the probiotic *Lactobacillus rhamnosus* showed reduced expression of GABA_Aα2_ mRNA in the prefrontal cortex (Bravo *et al*., 2011).

### (3) Hippocampus

The hippocampus plays an essential role in the generation and maintenance of cognitive spatial maps (O’Keefe & Dostrovsky, 1971). Although often not considered within the typical network comprising the social brain, it is becoming increasingly apparent that the hippocampus plays an important role in mammalian social cognition. For example, the hippocampus contributes to social recognition and social memory (Kogan, Franklandand, & Silva, 2000). Analogous to its role in navigating physical space, researchers have also recently uncovered hippocampal contributions to navigating ‘social’ space in humans (Tavares *et al*., 2015). In particular, the hippocampus tracks others in this social space based on their degree of affiliation or closeness to the self and the social status they possess (Taveras *et al*., 2015). Importantly, hippocampal abnormalities, including cellular changes and volumetric reduction, have also been linked to depression (MacQueen *et al*., 2003; Hastings *et al*., 2004; Stockmeier *et al*., 2004; Videbech & Ravnkilde, 2004; Rosso *et al*., 2005). As such, it is worth considering the possibility that some of the relationships between the microbiome and depression could be mediated by changes in hippocampal structure and function.

The effects of the microbiome on the rodent hippocampus are some of the most consistent in the microbiome–gut–brain field. For example, germ-free mice show reduced levels of hippocampal brain-derived neurotrophic factor (BDNF) and BDNF mRNA (Clarke *et al*., 2013; Diaz Heijtz *et al*., 2011; Sudo *et al*., 2004), a protein involved in neuroplasticity and memory (Greenberg *et al*., 2009). Furthermore, both prebiotics and probiotics increase hippocampal BDNF levels (Desbonnet *et al*., 2008; Savignac *et al*., 2013; Burokas *et al*., 2017).

Relative to normally colonised controls, germ-free status in mice impacts several aspects of dendritic morphology in the hippocampus (as well as the amygdala), including reduced dendritic length and a smaller number of branch points (Luczynski *et al*., 2016). Overall hippocampal dendritic spine density is also lower in germ-free mice, a reduction accounted for by reduced densities of stubby spines and mushroom spines (Luczynski *et al*., 2016). At the same time, germ-free mice also show greater total volume of certain hippocampal regions, such as CA2/3 (Luczynski *et al*., 2016). Evidence is also emerging that the microbiome regulates adult neurogenesis in the hippocampus (Möhle *et al*., 2016; Ogbonnaya *et al*., 2015). In particular, germ-free status in mice elevates hippocampal neuroproliferation that is not reversible by colonisation with a normal microbiome (Ogbonnaya *et al*., 2015). However, antibiotic exposure in adult mice supresses hippocampal neurogenesis, but this can be reversed *via* treatment with probiotics (Möhle *et al*., 2016).

There is much less evidence of a hippocampal association with the microbiome in humans, but subgroup analysis from one small study suggests that individuals with high levels of *Prevotella* spp. may have lower hippocampal volume, and also show reduced hippocampal activity in response to negative emotional images (Tillisch *et al*., 2017). Since activity in the hippocampus has been associated with emotional regulation (Phelps, 2004), reduced *Prevotella*-associated hippocampal activation in response to negative emotional stimuli may be a risk factor for certain psychiatric conditions (Tillisch *et al*., 2017), although of course such an interpretation is highly speculative (the result itself should be subject to replication, and the causal contribution of *Prevotella* should be assessed).

## V. MICROBIAL REGULATION OF SOCIAL SIGNALLING MOLECULES

**Fig. 5.**
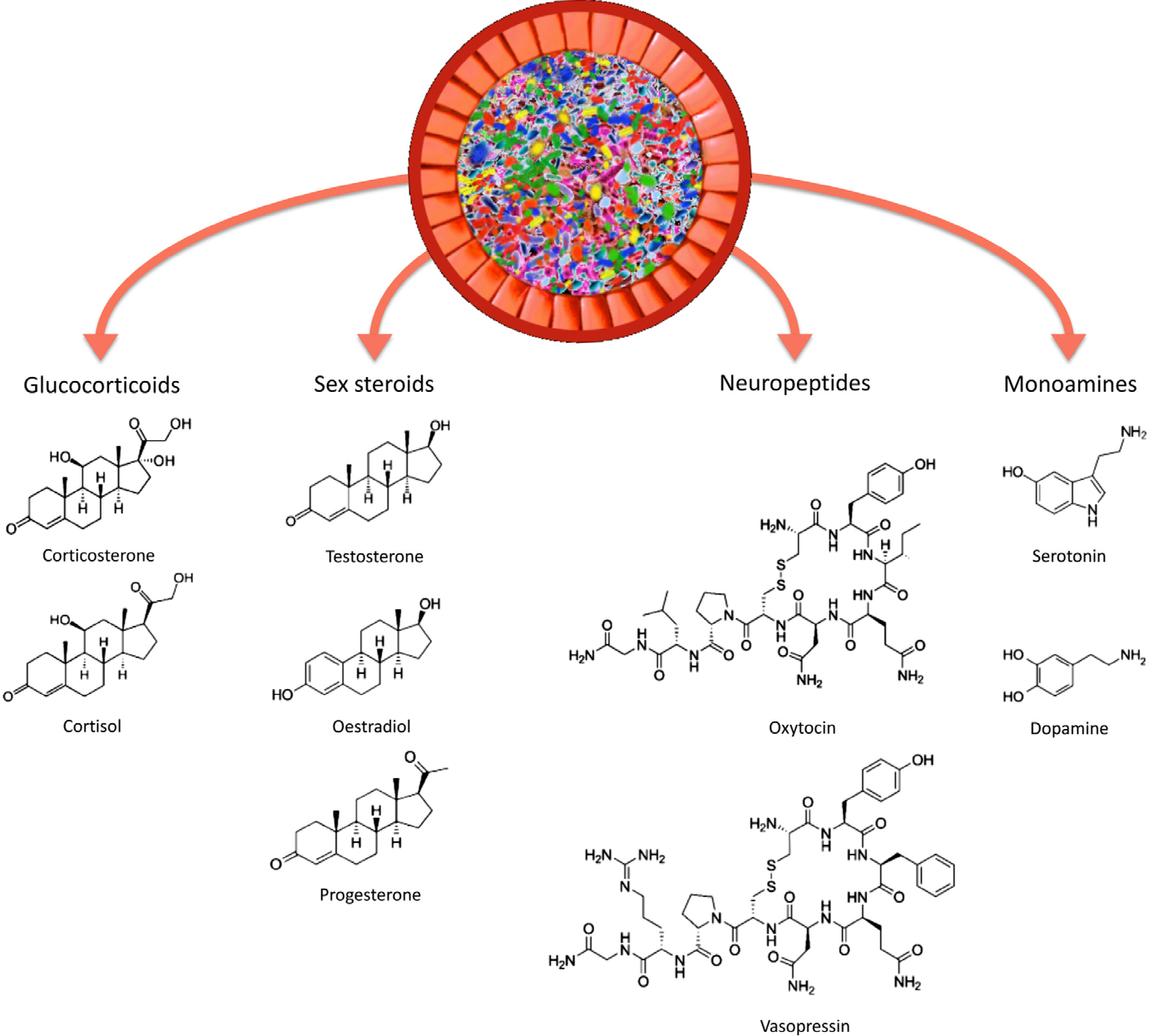
Microbial regulation of social signalling molecules. Gut microbes regulate the biosynthesis of a range of molecules that mediate social behaviour, including glucocorticoids such as corticosterone and cortisol, androgens such as testosterone, oestrogens such as oestradiol, progestogens such as progesterone, monoamines such as serotonin and dopamine, and neuropeptides such as oxytocin and arginine vasopressin. In addition to producing some of these molecules directly, gut microbes also alter their concentrations and bioavailability *via* interactions with host tissue, or by secreting enzymes that deconjugate signalling molecules into their active forms. There is evidence that gut bacteria can causally affect these signalling pathways. In addition, these signalling molecules may in turn influence the microbial communities of the gut, either directly, by affecting other functions (such as host immunity), as substrates used in microbial metabolism, or *via* microbial effects on host social behaviour which may influence the probability of socially transmitted microbes entering the gut. In the case of progestogens such as progesterone and monoamines such as serotonin, there is experimental evidence that these molecules can influence microbial populations directly.

In addition to modulating brain anatomy and physiology, the microbiome may also affect the central nervous system *via* the generation and regulation of a range of ‘social’ signalling molecules including glucocorticoids, sex steroids, neuropeptides, and monoamines (see Fig. 5). Microbial communities regulate the biosynthesis and bioavailability of several neurotransmitters that play important roles in animal social interaction. There has also been a steadily growing interest in microbial endocrinology in terms of the relationship between microbes and host neuroendocrine function (Lyte, 2014), and such microbe–hormone interactions could be relevant to social behaviour. For instance, the microbiome affects several steroids regulated by the hypothalamus, including along the hypothalamic–pituitary–adrenal (HPA) axis and hypothalamic–pituitary–gonadal (HPG) axis.

There are at least three non-mutually exclusive pathways by which microbes regulate the biosynthesis and bioavailability of these signalling molecules. First, these molecules may be generated as by-products of bacterial metabolism. For instance, *Lactobacillus* and *Bifidobacterium* secrete GABA, *Lactobacillus* secretes acetylcholine, *Escherichia* and *Bacillus* secrete norepinephrine, and *Bacillus* and *Serratia* secrete dopamine (Lyte, 2011). Second, bacterial metabolites such as SCFAs and secondary bile acids can interact with host cells that regulate the production of signalling molecules. Third, signalling molecules can be converted into their active forms *via* bacterially mediated enzymatic deconjugation. In the examples that follow, we describe instances of all three processes.

These signalling molecules also vary in their brain-penetrant properties, with some readily able to cross the blood–brain barrier (e.g. glucocorticoids and sex steroids), while others are thought to be unable to do so (e.g. oxytocin). Overall, these molecules may exert their behavioural effects by entering the brain directly (if the molecule or its precursor can cross the blood–brain barrier), *via* effects on the immune system, or by modulating activity of the vagus nerve (Johnson & Foster, 2018). They may also perhaps exert their behavioural effects by modulating the activity of the proximal synapses of the enteric nervous system that innervates the gut, changes that may then be relayed to the brain (Sarkar *et al*., 2016; Johnson & Foster, 2018).

### (1) Glucocorticoids

The gut microbiome influences concentrations of endogenous steroids, including glucocorticoids such as cortisol and corticosterone, which are the hormonal end-products of the HPA axis. The primary physiological function of glucocorticoids is glucose metabolism, a process that prepares the body for action by releasing energy. Importantly, once glucocorticoids are released into systemic circulation, they are also able to cross the blood–brain barrier, and can therefore interact directly with the central nervous system (Pardridge & Mietus, 1979). At the psychological level, glucocorticoid release is tightly coupled with the experience of fear and anxiety (Dickerson & Kemeny, 2004). The elevatation of glucocorticoids is considered to be one of the physiological hallmarks of stress. Hyperactivity of the HPA axis in humans predicts behaviours such as social avoidance (Roelofs *et al*., 2009), which have implications for social interaction. Similarly, pharmacologically elevating corticotropin-releasing factor in rodents enhances anxiety and supresses normal social interaction (Dunn & File, 1987).

The effect of the microbiome on the development of the HPA axis, and therefore its influence on the host’s stress response, has become an important area of investigation (de Weerth, 2017). For instance, germ-free rodents consistently show elevated corticosterone levels in response to stress compared with normally colonised animals (Sudo *et al*., 2004; Neufeld *et al*., 2011; Crumeyrolle-Arias *et al*., 2014). Ingestion of probiotics and prebiotics has been noted to reduce levels of circulating glucocorticoids in both humans and rodents, and is also associated with decreased anxiety (Bravo *et al*., 2011; Messaoudi *et al*., 2011; Schmidt *et al*., 2015; Allen *et al*., 2016; Burokas *et al*., 2017).

### (2) Sex steroids

Gut microbes are also associated with the activity of host sex steroids such as androgens, oestrogens, and progestogens, the hormonal end-products of the HPG axis. Like glucocorticoids, sex steroids are capable of crossing the blood–brain barrier and can therefore bind directly to neurons in the brain (Pardridge & Mietus, 1979). It has been known for several decades that the microbiome regulates the bioavailability of endogenous steroids, as early studies found that germ-free rats produced very small quantities of steroids compared to normally colonised rats (Eriksson, Gustafsson, & Sjövall, 1969). Germ-free status was also found to interfere with normal reproduction in both males and females, and these effects were reversed by microbial colonisation (Shimizu *et al*., 1998). Technological advances have resulted in more fine-grained studies, and many of the microbial effects on these molecules have been investigated within the last decade.

#### (a) Androgens

Androgens are a major class of steroids that regulate male sexual development, exerting a variety of important physiological and psychological effects. They are also present in much smaller quantities in females, but their role in female biology and behaviour is generally less well understood compared to males. The primary androgen is testosterone, an end-product of the HPG axis. Others include androstenedione, dehydroepiandrosterone, and dihydrotestosterone. In males, rising testosterone levels associated with adolescence trigger sexual development, spermatogenesis, and the development of secondary sexual characteristics (Mooradian, Morley, & Korenman, 1987; Hau, 2007; Walker, 2011, 2009). From the perspective of animal sociality, testosterone controls mating and reproductive behaviour, especially in males, and is implicated in the motivation for status-seeking, including in humans (Mazur, 1985; Mazur & Booth, 1998; Archer, 2006; Eisenegger, Haushofer, & Fehr, 2011).

Male germ-free mice show markedly lower serum testosterone concentrations compared to normally colonised male conspecifics, while female germ-free mice show the opposite pattern (Markle *et al*., 2013). The transplantation of microbes from adult males into pre-adolescent female recipients (*via* faecal transfer) increases testosterone concentrations in the recipients (Markle *et al*., 2013). Similarly, researchers have found that germ-free status is associated with lower levels of both circulating gonadotropins and intratesticular testosterone concentrations, as well as reduced integrity of the blood–testis barrier, which protects the gonads from many peripheral influences such as proinflammatory factors (Al-Asmakh *et al*., 2014). The impairment in blood-testis barrier integrity in germ-free mice was associated with reduced expression of cell adhesion proteins, while colonisation with *Clostridium tyrobutyricum* ameliorated the expression of cell adhesion proteins and restored the integrity of the blood–testis barrier (Al-Asmakh *et al*., 2014). Furthermore, ageing mice fed *Lactobacillus reuteri* show higher concentrations of serum testosterone and enhanced rates of spermatogenesis (Poutahidis *et al*., 2014). Moreover, relative to controls, mice treated with *Lactobacillus reuteri* display both morpho-morphological changes (as measured by greater testis size) and cellular changes (as measured by a proliferation of testosterone-producing Leydig cells) (Poutahidis *et al*., 2014). Together, these findings point to a causal role of the gut microbiome in the biosynthesis or regulation of testosterone and testicular morphology and function across the lifespan, suggesting that the microbiome may therefore influence some aspects of reproduction and reproductive behaviour.

#### (b) Oestrogens

The microbiome also influences endogenous concentrations of oestrogens, which are an important group of ‘female’ reproductive steroids (they are also present in smaller quantities in males). They include oestradiol (the primary oestrogen), oestrone, and oestriol. Oestrogens regulate the maturation and maintenance of the female reproductive system (McCarthy, 2008; Colvin & Abdullatif, 2013). Compared to testosterone, much less research has been done on the social and behavioural correlates of oestrogens, although there is some evidence that oestradiol drives female competition and status-seeking behaviour in humans (Knight & Mehta, 2014; Stanton & Edelstein, 2009; Stanton & Schultheiss, 2007).

The microbiome plays an important role in the availability of oestrogens (Flores *et al*., 2012; Fuhrman *et al*., 2014), and researchers have developed the concept of the ‘estrobolome’, or the total collection of bacterial genes that encodes products capable of metabolising oestrogens (Plottel & Blaser, 2011). Disturbances in the estrobolome are thought to be associated with breast cancer (Kwa *et al*., 2016). A significant proportion of oestrogen molecules are hepatically conjugated with glucuronide or sulphate, rendering them inactive, and their resultant polarity allows for re-entry into the lumen and subsequent excretion (Kwa *et al*., 2016). This phenomenon potentially prevents a substantial quantity of oestrogens from exerting physiological effects. However, several bacteria intervene in this process. For example, some bacteria can influence the concentration of active oestrogen through their capacity to encode enzymes such as *β*-glucuronidase and *β*-glucosidase, which deconjugate oestrogen molecules (Dabek *et al*., 2008; Kwa *et al*., 2016). This deconjugation of oestrogen molecules into their active forms enables their intestinal reabsorption and return to circulation. Thus, gut microbes can enhance the bioavailability of oestrogens beyond the host’s intrinsic capacity. In humans, some early studies found that antibiotic treatment increased the presence of conjugated oestrogens in faeces, suggesting that antibiotics could suppress microbially mediated deconjugation in the gut, an effect observed in both females (Adlercreutz *et al*., 1975; Martin *et al*., 1975) and males (Hämäläinen, Korpela, & Adlercreutz, 1987). While of course these results could be attributable to off-target effects of antibiotics, the close association between the microbiome and host oestrogens does suggest that antibiotics may exert a potent effect on the bioavailability of oestrogens *via* loss of microbial enzymes necessary for the deconjugation of oestrogen molecules.

#### (c) Progestogens

Researchers have also recently detected microbiome–progestogen associations. Like oestrogens, progestogens are ‘female’ steroid hormones that contribute to female reproductive processes (Colvin & Abdullatif, 2013). However, like oestrogens, progestogens are also present in males in small quantities and contribute to male biology. The primary progestogen is progesterone, and others include 16*α*-hydroxyprogesterone, 3*β*-dihydroprogesterone, and 5*α*-dihydroprogesterone. Progesterone is involved in female reproduction and related processes, including regulation of the menstrual cycle, maintenance of pregnancy, inhibition of milk production during pregnancy, and breast development. At the behavioural level, it has been suggested that progesterone is involved in human social bonding and affiliation (Brown *et al*., 2009; Fleischman, Fessler, & Cholakians, 2015; Gangestad & Grebe, 2017; Schultheiss, Wirth, & Stanton, 2004; Wirth & Schultheiss, 2006).

The host microbiome changes continuously over the course of pregnancy, with particularly large differences between the first and third trimesters (Koren *et al*., 2012). Notably, recent work has found substantial progesterone-associated changes in the microbiomes of both humans and mice (Nuriel-Ohayon *et al*., 2019). Specifically, the relative abundance of *Bifidobacterium* spp. increases in the later stages of pregnancy (Nuriel-Ohayon *et al*., 2019). Subsequent analysis found that the presence of progesterone sharply elevated the relative abundance of *Bifidobacterium* spp. both *in vivo* and *in vitro*, suggesting that progesterone is able to alter microbial composition (Nuriel-Ohayon *et al*., 2019).

### (3) Neuropeptide hormones

#### (a) Oxytocin

Oxytocin is a neuropeptide hormone produced mainly in the hypothalamus. It plays an evolutionarily conserved role in mating and reproductive behaviour (Garrison *et al*., 2012; Feldman, 2017). Oxytocin and oxytocin-like molecules perform these functions in animals ranging from invertebrates such as nematodes (Garrison *et al*., 2012; Elphick, Mirabeau, & Larhammar, 2018) to humans (Feldman, 2017). At the psychological level, oxytocin plays a prominent role in mammalian social attachment, beginning with the mother–infant bond, followed by bonds with other social partners as the mammal matures (Feldman, 2017). There has also been a great deal of interest in the prosocial effects of oxytocin, particularly following the finding that exogenously administered oxytocin promotes interpersonal trust (Kosfeld *et al*., 2005). However, subsequent studies have failed to replicate this result (Lane *et al*., 2015; Nave, Camerer, & McCullough, 2015), and at the very least, the oxytocin → trust relationship is not as straightforward as originally anticipated. Moreover, it is currently believed that it is not possible for peripheral oxytocin to cross the blood–brain barrier to exert effects on the central nervous system (Ermisch *et al*., 1985; Leng & Ludwig, 2016).

A range of studies suggests that the gut microbiome can influence oxytocin signalling (Erdman & Poutahidis, 2016). Antibiotic administration reduces hypothalamic oxytocin levels in mice, alongside depleting microbial populations (Desbonnet *et al*., 2015). As discussed earlier, the offspring of mice fed high-fat diets during pregnancy display significant social impairments and have fewer hypothalamic oxytocin-expressing neurons, attributable to maternal diet-induced differences in their gut bacteria (Buffington *et al*., 2016). Moreover, early ingestion of the probiotic *Lactobacillus reuteri* in the offspring restored the number of oxytocin-expressing neurons in the mice and attenuated the social deficits.

Treatment with *Lactobacillus reuteri* also increased the number of oxytocin-positive neurons and their oxytocin expression in the paraventricular nucleus of *Shank3*-knockout mice, which otherwise had fewer such neurons in this brain region (Sgritta *et al*., 2019). Furthermore, the social benefits of *Lactobacillus reuteri* are dependent on oxytocinergic signalling in the ventral tegmental area. Specifically, *Shank3*-knockout mice lacking oxytocin receptors in dopamine neurons did not show improvements in their impaired social behaviour, and also did not show normal levels of long-term potentiation in the ventral tegmental area following social interaction (Sgritta *et al*., 2019). As such, the capacity of this probiotic to exert effects on host social behaviour appears to depend on the integrity of the oxytocin signalling system. As mentioned earlier, vagotomy abolished the beneficial effects of *Lactobacillus reuteri*, suggesting that the vagus nerve mediates this relationship. Beyond these central effects, administration of *Lactobacillus reuteri* to mice has also been found to upregulate plasma oxytocin levels *via* the vagus nerve (Poutahidis *et al*., 2013).

Interestingly, *Lactobacillus reuteri* appears to increase both oxytocin and testosterone signalling, and also suppresses glucocorticoid signalling (Poutahidis *et al*., 2013; Buffington *et al*., 2016; Varian *et al*., 2017). The mechanism by which a single probiotic exerts effects on both neuropeptides and steroids remains unknown, although one possibility is that these effects occur *via* changes in the immune system. Also, given the involvement of the hypothalamus in these signalling pathways, and since the gut microbiome has been shown to affect the hypothalamus (Buffington *et al*., 2016), it is plausible that *Lactobacillus reuteri* produces these effects by modulating hypothalamic function.

#### (b) Arginine vasopressin

Arginine vasopressin (vasopressin) is a neuropeptide hormone that is structurally similar to oxytocin, and, like oxytocin, is produced mainly in the hypothalamus. Amongst the primary physiological functions of vasopressin are the control and regulation of the organism’s water balance and cardiovascular function (Share, 1988; Nielsen *et al*., 1995). Like oxytocin, systemic vasopressin is unable to cross the blood–brain barrier. At the psychological level, vasopressin has been implicated in maternal behaviour. For example, in rodents, vasopressin promotes maternal aggression towards intruders (Bosch & Neumann, 2010). Central vasopressin has also been found to be positively associated with sociability in monkeys, with some evidence of a similar association in humans as well (Parker *et al*., 2018). In general, the microbiome–vasopressin relationship has not received as much attention as the microbiome–oxytocin relationship. However, some interesting patterns have been observed that suggest this may be a worthwhile area of investigation. For instance, the administration of antibiotics to mice reduces hypothalamic vasopressin expression (Desbonnet *et al*., 2015). There is also recent, intriguing evidence from rats that deletion of the *Avp* gene (which controls vasopressin expression in the brain) leads to sex-specific changes in the composition of the microbiome, including an increase in *Lactobacillus* spp. in males (Fields *et al*., 2018).

### (4) Monoamines

#### (a) Serotonin

The indolamine serotonin (5-hydroxytryptamine) is a metabolite of the essential amino acid tryptophan. Serotonin regulates a variety of physiological processes in the host, including normal gastrointestinal, cardiovascular, and excretory functions (Berger, Gray, & Roth, 2009). In terms of host psychological processes, the serotonergic system is implicated in emotion regulation, social cognition, and social interaction (Young & Leyton, 2002; Canli & Lesch, 2007). Serotonergic signalling is also implicated in social dominance and aggression across the animal kingdom (Nelson & Chiavegatto, 2001). Serotonergic dysfunction has also been linked to psychiatric disorders such as depression (Owens & Nemeroff, 1994). However, researchers are discovering that the aetiology of depression extends well beyond serotonergic disruption, and there is increasing evidence that clinical depression is a highly heterogeneous disorder with multiple, intertwined aetiologies linked to alterations in brain plasticity and monoamine functions in general, as well as disturbances in the immune system and the HPA axis (Miller & Raison, 2016; Pariante, 2017; Levy *et al*., 2018).

There has been a great deal of interest in the association between the microbiome, tryptophan metabolism, and the regulation of host serotonergic signalling (O’Mahony *et al*., 2015). Compared to normally colonised mice, male germ-free mice were found to have substantially higher levels of plasma tryptophan, but substantially lower levels of plasma serotonin, suggesting that the absence of gut microbes impairs the peripheral conversion of tryptophan into serotonin (Wikoff *et al*., 2009; Clarke *et al*., 2013). Microbial transfer *via* faecal transplants from normally colonised mice to germ-free mice is sufficient to increase peripheral serotonin concentrations within a few days of colonisation (Hata *et al*., 2017). On the other hand, male (but not female) germ-free mice also have significantly increased concentrations of serotonin in the hippocampus (Clarke *et al*., 2013) and increased serotonin turnover in the striatum (Diaz Heijtz *et al*., 2011). This gives rise to an important conceptual puzzle: why – and through what mechanism – does the absence of gut bacteria increase central serotonin levels (Clarke *et al*., 2013) and its turnover (Diaz Heijtz *et al*., 2011), but decrease peripheral serotonin levels (Clarke *et al*., 2013; Wikoff *et al*., 2009)? Furthermore, are these changes related to one another, and do they occur *via* a compensatory mechanism? Two of these studies (Clarke *et al*., 2013; Wikoff *et al*., 2009) used Swiss Webster mice, and therefore species-level variations in genetic background are less likely to account for such differences between peripheral and central serotonin levels. One possibility relates to the potential role of serotonin in meeting the brain’s energy demands. In particular, researchers have recently hypothesised that one of the primary functions of serotonin in the brain is to support and regulate its energetic and metabolic requirements, including in the hippocampus (Andrews *et al*., 2015). If this is correct, then the enhanced hippocampal serotonin concentrations in germ-free mice (Clarke *et al*., 2013) might be attributable to central-level differences in energy demands between germ-free and normally colonised mice. One of the key roles of the microbiome is the regulation of host peripheral metabolism (Turnbaugh *et al*., 2006; Nicholson *et al*., 2012; Perry *et al*., 2016), and it could be plausible that the microbiome also influences metabolism in the central nervous system. At the very least, this hypothesis warrants experimental investigation.

The mechanisms underlying serotonin differences in germ-free and normally colonised animals are still under investigation. One possibility is that the microbes themselves generate a considerable quantity of serotonin. Indeed, bacteria including species of *Candida, Enterococcus, Escherichia,* and *Streptococcus* are capable of secreting serotonin directly (Lyte, 2011), although it is unknown whether, and to what extent, this occurs in the gut environment (Johnson & Foster, 2018). A recent investigation found that indigenous sporeforming bacteria (and particularly those from the genus *Clostridium*) can regulate the host’s gut-based serotonin biosynthesis (Yano *et al*., 2015). These bacteria produce metabolites such as SCFAs that promote serotonin production by the host’s enterochromaffin cells (Reigstad *et al*., 2015; Yano *et al*., 2015). Thus, it may be that the majority of bacterial contributions to host serotonin arise from bacterially derived metabolites regulating the production of serotonin by the host’s enterochromaffin cells, rather than from serotonin directly produced by the bacteria themselves. Furthermore, recent research also suggests that much of the luminal serotonin in germ-free mice is conjugated with glucuronide and is rendered biologically inactive (Hata *et al*., 2017). Bacterially derived enzymes deconjugate glucuronidated serotonin molecules, increasing the total amount of bioavailable serotonin in the lumen (Hata *et al*., 2017).

Importantly, as systemic serotonin is thought to be unable to cross the blood–brain barrier, it is currently unclear whether microbially derived peripheral serotonin is able to affect the activity of the central nervous system directly. The general implications of free luminal serotonin are presently unclear. However, a proportion of this serotonin may be used in bacterial metabolism. In particular, there is evidence that serotonin may promote the growth of some bacteria (Roshchina, 2016). If serotonin is able to stimulate the growth of particular bacterial taxa, then deconjugating serotonin molecules in the lumen into free serotonin might directly enhance the fitness of these bacteria by enhancing their growth and reproduction. A recent study has shown that enhancing serotonin levels using pharmacological and genetic manipulations substantially increases the abundance of *Clostridia* spp. and *Turicibacter* spp., suggesting that some gut bacteria are able to detect and respond to serotonin as a growth factor (Fung *et al*., 2019). In particular, *Turicibacter sanguinis* was found to possess genetic adaptations that enable serotonin importation. This helps the bacterium gain an advantage over other bacteria in colonising the gastrointestinal tracts of mice supplemented with serotonin. On the other hand, the drug fluoxetine (a selective serotonin reuptake inhibitor frequently used as an antidepressant) reduces the capacity of *Turicibacter sanguinis* to utilise host serotonin by inhibiting its serotonin-importer system, thereby reducing its competitive advantage. Interestingly, when mice were monoassociated with *Turicibacter sanguinis*, supplementation with serotonin did not enhance bacterial growth. This suggests that serotonin supplementation is only advantageous when *Turicibacter sanguinis* faces competition from other microbes in colonising the gut (Fung *et al*., 2019).

#### (b) Dopamine

Gut microbes have also been noted to influence the concentrations of the endogenous catecholamine dopamine (3,4-dihydroxyphenethylamine), which is synthesised from its precursor, the amino acid levodopa (l-3,-4-dihydroxyphenylalanine), which is itself synthesised from tyrosine (4-hydroxyphenylalanine) (Nagatsu, Levitt, & Udenfriend, 1964; Shiman, Akino, & Kaufman, 1971). Levodopa (which is also used pharmacologically to treat Parkinson’s disease) occurs naturally in the body. It is able to cross the blood–brain barrier, where it is converted into dopamine. Peripheral dopamine, however, cannot cross the blood–brain barrier.

The physiological roles of dopamine include motor control and coordination (Howe & Dombeck, 2016), as well as the regulation of cardiovascular and renal function (Goldberg, 1972). At the psychological level, dopamine is best known for its role in the reward system, where it plays a fundamental part in reward learning and prediction (Schultz, 2002). Central dopaminergic signalling in brain regions such as the striatum and ventral tegmental area is also thought to facilitate social bonding by enhancing the reward value of social interaction (Feldman, 2017), and some evidence from dopamine receptor genetics suggests a role for dopamine in the size and maintenance of human social networks (Pearce *et al*., 2017).

Comparisons between germ-free and normally colonised mice have found that germ-free specimens show reduced levels of luminal dopamine (Velagapudi *et al*., 2010; Asano *et al*., 2012). Moreover, a substantial proportion of luminal dopamine in germ-free mice was conjugated with glucuronide or sulphate and biologically inactive, whereas the reverse pattern was observed in normally colonised mice (Asano *et al*., 2012). Both conventionalisation with a normal microbiome and colonisation with *Clostridium* spp. increased levels of dopamine and *β*-glucuronidase. Moreover, comparisons between mice colonised with *Escherichia coli* (capable of secreting *β*-glucuronidase) and mice colonised with an *Escherichia coli* mutant (incapable of producing *β*-glucuronidase) revealed that luminal dopamine in mice colonised with the mutant strain (in which *β*-glucuronidase production was suppressed) was conjugated and biologically inactive, suggesting a causal role for bacterially derived *β*-glucuronidase activity in regulating luminal dopamine availability.

Another example of microbial metabolism influencing the availability of dopamine in the gut is a recent study reporting that gut bacteria metabolise exogenously administered levodopa into dopamine, and then convert dopamine into *m*-tyramine (Maini Rekdal *et al*., 2019). Specifically, *Enterococcus faecalis* converts levodopa into dopamine *via* a decarboxylation reaction, and *Eggerthella lenta* converts dopamine into *m*-tyramine *via* a dihydroxylation reaction (Maini Rekdal *et al*., 2019). The implications of this phenomenon for central dopamine levels are presently unclear. It may be that gut microbes such as *Enterococcus faecalis* convert naturally occurring levodopa to dopamine which might therefore lead to reduced central dopamine availability in the brain (since dopamine cannot cross the blood–brain barrier, unlike its precursor levodopa). Further research is necessary to examine whether the natural conversion of levodopa to dopamine by gut bacteria has a significant effect on dopamine levels in the brain.

Researchers have also observed direct relationships between the microbiome and central dopamine activity. For instance, studies have found an increased concentration of brain dopamine in germ-free mice (Matsumoto *et al*., 2013; Nishino *et al*., 2013). Compared with normally colonised conspecifics, germ-free mice also showed elevated hippocampal expression of mRNA encoding D1, a key dopamine receptor (Diaz Heijtz *et al*., 2011), as well as elevated levels of striatal dopaminergic turnover (Diaz Heijtz *et al*., 2011). However, enhanced central dopamine turnover has not consistently been observed. For instance, one study comparing germ-free and normally colonised rats found *lower* dopaminergic turnover in the frontal cortex, hippocampus, and striatum in germ-free specimens (Crumeyrolle-Arias *et al*., 2014).

Clearly, while additional studies are needed to clarify the role of the microbiome in dopaminergic turnover, the available data at least suggest that the microbiome can influence central dopaminergic signalling. Moreover, Sgritta *et al.* (2019) found that the probiotic-induced rescue of social deficits in autistic-type mice required the presence of oxytocin receptors on dopamine neurons in the ventral tegmental area. Thus, alongside oxytocinergic signalling, dopaminergic signalling also appears to be necessary for the benefits of *Lactobacillus reuteri*.

## VI. MICROBIAL REGULATION OF GENE EXPRESSION AND EPIGENETIC PROCESSES IN THE SOCIAL BRAIN

There is growing interest in microbial contributions to social behaviour at the level of host gene expression and epigenetic mechanisms (Stilling *et al*., 2014). In particular, if the microbiome is affecting brain morphology and function and hormonal and neurotransmitter signalling, then it can be expected that the microbiome also influences host gene expression. In this regard, research is now revealing that the microbiome can modulate gene activity relevant to sociality.

### (1) Neuroanatomical distribution of gene expression

Relative to normally colonised mice, germ-free mice show extensive dysregulation in networks of micro-ribonucleic acids (miRNAs) in the amygdala and prefrontal cortex, both key regions of the social brain (Hoban *et al*., 2017). Some, but not all, of these aberrations were attenuated by colonisation with a normal microbiome (Hoban *et al*., 2017). Furthermore, gene expression profiling in the amygdalas of germ-free mice (compared to normally colonised mice) revealed elevated expression of transcription factor genes (*Fos, Egr2, Nr4a1*) and the genes *Arc* and *Homer1* that are indices of increased BDNF signalling and neuronal activation, respectively (Stilling *et al*., 2015).

The absence of microbes is associated with other pre- and post-transcriptional events including differential exon splicing and editing of mRNAs that ultimately sculpt changes in neuronal function (Hoban *et al*., 2017, 2018; Stilling *et al*., 2018). These studies found that the expression of transcription factor genes and genes involved in neuronal activity were elevated in the amygdalas of both germ-free and normally colonised mice that had recently engaged in social interactions, suggesting that social interaction rapidly affects amygdalar gene expression. Importantly, however, the amygdalar neurons of germ-free animals displayed higher rates of alternative splicing (Stilling *et al*., 2018), a process that expands the number of proteins that could otherwise be encoded by a given number of genes, ultimately increasing the range of biological functions those genes can perform. Furthermore, in germ-free mice, there is enhanced expression of genes regulating cholinergic and dopaminergic neurotransmission, which are associated with amygdalar learning (Hoban *et al*., 2018).

These changes in splicing and expression observed in germ-free animals likely represent aberrant alterations in host genetics as a result of the absence of microorganisms. For example, we might speculate that these variations in splicing and gene expression reflect compensatory processes initiated by the host, such that functions which would otherwise be supported by microbiome-related activity can be fulfilled in the absence of microbes. Another possibility is that this increased alternative splicing is maladaptive, and is kept in check by microbiome-related processes.

Overall, germ-free mice show deficits in social development (Desbonnet *et al*., 2014; Buffington *et al*., 2016; Stilling *et al*., 2018) and there is evidence of an association between aberrant gene expression and neuronal function in the amygdalas of germ-free animals in response to social challenges (Stilling *et al*., 2018). For instance, an enrichment of RNA-splicing genes – but not those involved in mitogen-activated protein kinase (MAPK) cell signalling pathways – was observed in germ-free mice following social interaction (Stilling *et al*., 2018).

Based on these observations, it is reasonable to speculate that perturbations of the microbiome in normally colonised animals may impact their social behaviour *via* changes in gene and protein expression. For example, antibiotic-induced dysbiosis in mice reduced both social recognition and hippocampal BDNF levels, but elevated the expression of the BDNF receptor, tropomyosin receptor kinase B (TrkB) (Guida *et al*., 2018). Furthermore, ingestion of the probiotic *Lactobacillus casei* normalised both central BDNF levels and social recognition memory, although TrkB densities remained elevated (Guida *et al*., 2018). However, it should be noted that the design of this study did not permit examination of the possibility that TrkB density simply requires longer to return to normal. The capacity of this single-strain probiotic to rescue deficits in social recognition from antibiotic-induced dysbiosis suggests the possibility that discrete changes in a complex microbial community may be able to affect brain function, although of course these effects may occur *via* other pathways, such as modulation of the immune system.

### (2) Epigenetic effects of glucocorticoids

It remains unclear what causes changes in central gene expression. One possibility is that the peripheral neuroendocrine stress response, mediated by the HPA axis, is a key link between gut microbes and host behaviour (Cryan & Dinan, 2012; Foster & McVey Neufeld, 2013; de Weerth, 2017). Glucocorticoids (which are elevated in circulation during stressful events) enter the brain and bind to glucocorticoid receptors which are abundantly expressed in the hippocampus and amygdala. Within the cell nucleus, the ligand-bound glucocorticoid receptors can affect transcription by direct high-affinity binding to glucocorticoid response elements found either in the promoters or the intragenic regions of glucocorticoid target genes (Tan & Wahli, 2016). Therefore, a heightened stress response is likely to result in changes in activity of hippocampal and amygdalar neurocircuitry, with subsequent changes in social behaviour. Consistent with this supposition, the activation of hippocampal glucocorticoid receptors has been shown to enhance contextual fear memory via elevation of BDNF signalling (Revest *et al*., 2014). This result corroborates the observation that germ-free mice have increased amygdalar BDNF levels, which is accompanied by an exaggerated stress response (Sudo *et al*., 2004). Of course, the mechanisms *via* which the microbiome can regulate HPA-axis activity are still being elucidated, and potentially include microbiome interactions with the gut immune system and the enteric nervous system (e.g. through direct contact or neurotransmitters secreted by bacteria, as described earlier in Section V).

### (3) Epigenetic effects of microbial metabolites

Some of the microbial effects on host physiology may also be orchestrated by the metabolites that the microbes generate from breaking down complex dietary carbohydrates in the host’s diet. For example, the fermentation of indigestible carbohydrates by the gut microbiome produces SCFAs (Koh *et al*., 2016; Sarma *et al*., 2017), which can then enter systemic circulation and modulate sympathetic nervous system activity (Kimura *et al*., 2011). Microbially generated SCFAs include acetate, butyrate, and propionate. Of these, most of the butyrate is readily absorbed by epithelial cells of the colon where it is utilised as an energy source, and promotes anti-inflammatory responses (Koh *et al*., 2016). While propionate and a proportion of the acetate bind to specific receptors in the gut and initiate the release of gut hormones (Koh *et al*., 2016), the majority of acetate is taken up into the vascular system and distributed throughout the organs, including the brain (Koh *et al*., 2016; Perry *et al*., 2016). Glial cells can use acetate as a source of energy, but more importantly, this SCFA can exert epigenetic effects through the inhibition of histone deacetylases (Rae *et al*., 2012). That is, acetate promotes the process that allows the transcription of genes to occur. More specifically, acetate is an inhibitor of histone deacetylases that remove acetate groups from genomic DNA and hinder the dissociation of the doublestranded molecule that must occur prior to gene transcription (Kasubuchi *et al*., 2015). Thus, in general, the inhibition of histone deacetylases increases gene expression. Like acetate, butyrate is also a potent histone deacetylase inhibitor, and accordingly may impact gene expression in the gut since this is where it is largely absorbed (Koh *et al*., 2016). However, since butyrate can cross the blood–brain barrier, its epigenetic effects may also extend to the brain.

In rats, both the oral administration of acetate and the intake of bifidogenic oligosaccharides (prebiotics) can increase the circulating concentrations of acetate, as well as the expression of genes encoding central glutamate N-methyl-D-aspartate (NMDA) receptor subunits and BDNF (Savignac *et al*., 2013; Gronier *et al*., 2018). Prebiotic feeding has also been shown to enhance the function of brain NMDA receptors, and improve cognitive flexibility in rats (Gronier *et al*., 2018). These findings are consistent with an earlier study showing that oral acetate supplementation rescued impairments in NMDA receptor function and, importantly, was associated with the inhibition of histone deacetylase activity (Singh *et al*., 2016). Therefore, one hypothesis is that acetate is a mediator of the procognitive effects of prebiotics, although this has yet to be formally tested. With regard to social behaviour, one investigation demonstrated that the inhibition of histone deacetylase activity in Syrian hamsters exacerbated behavioural responses to social stress, suggesting that epigenetic gene silencing may be favourable for the maintenance of normal social interactions (McCann *et al*., 2017). However, the inhibitor in this instance was sodium butyrate that was systemically or centrally administered at pharmacological doses (McCann *et al*., 2017), and therefore was not representative of the quantity and anatomical distribution of this SCFA when it is derived from the gut microbiome.

These findings collectively suggest the effects of the microbiome on host RNA biology and post-transcriptional processes, and provide evidence of potential microbial contributions to the genetic basis of social behaviour.

## VII. THE MICROBIOME AND SOCIAL OLFACTORY SIGNALS

The olfactory system plays an important role in conveying and detecting social information across the animal kingdom (Steiger, Schmitt, & Schaefer, 2011). The olfactory system participates in a variety of social processes, including territorial marking, discriminating between social groups, kin recognition, and mate detection and attraction. As just one example, in spotted hyaenas (*Crocuta crocuta*) a subcaudal gland secretion known as hyaena ‘paste’ relays a range of social information used for intra-specific signalling and communication (Drea *et al*., 2002a, 2002b; Burgener *et al*., 2009), including as a marker of social rank (Burgener *et al*., 2009).

### (1) The fermentation hypothesis

The fermentation hypothesis proposes that olfactory signals are the products of bacterial metabolism which the host exploits for chemical communication (Albone *et al*., 1974; Albone & Perry, 1976). These bacterially produced odourants may be generated in dedicated scent glands, and can be present in faeces, urine, or other secretions. Researchers are now finding, consistent with the fermentation hypothesis, that bacterial metabolism generates a range of odourants which communicate important social information, including sex, kinship, fertility, lactation status, health, and group membership *via* the olfactory system (Lizé, McKay, & Lewis, 2013; Ezenwa & Williams, 2014; Archie & Tung, 2015; Vuong *et al*., 2017; Bienenstock, Kunze, & Forsythe, 2018; Carthey, Gillings, & Blumstein, 2018).

### (2) A microbiome–olfaction–behaviour pathway?

Researchers have recently suggested that the microbiome–gut–brain axis may entail an underappreciated olfactory component – in other words, a microbiome–olfaction–behaviour pathway (Bienenstock *et al*., 2018). This olfactory component comprises the system of olfactory receptors and odourants, the molecules that bind to them. Olfactory receptors are widely distributed in the body. They are encoded by extensive multigene families, and are evolutionarily conserved across the animal kingdom. For example, the olfactory receptor multigene family comprises approximately 100 genes in catfish (Ngai *et al*., 1993), over 900 genes in mice (Godfrey, Malnic, & Buck, 2004), and over 300 genes in humans (Malnic, Godfrey, & Buck, 2004). In addition to the classical odour receptors, two new types of receptors have also been found to be involved in olfaction: trace-amine associated receptors and formyl peptide receptors (Bienenstock *et al*., 2018).

Importantly, host-associated microbes are capable of generating a number of odourants that bind to these receptors (i.e. classical odourant receptors, trace-amine associated receptors, and formyl peptide receptors), thereby modulating host tissue (Bienenstock *et al*., 2018). As such, some of the behavioural effects of the microbiome may be mediated by this broadly expressed system of olfactory receptors (Bienenstock *et al*., 2018). This microbiome–olfaction coupling may make larger contributions to host social behaviour than currently appreciated.

### (3) Insects

A relatively well-established body of research demonstrates that host-associated bacteria influence chemosignalling and communication between conspecifics by modulating odour profiles in insects. Bacterial effects on individual or colony-level chemical profiles, with subsequent effects on behaviour, have been observed across a range of insects, including ants (*Acromyrmex echinatior*; Dosmann, Bahet, & Gordon, 2016), cockroaches (*Blattella germanica*; Wada-Katsumata *et al*., 2015), fruit flies (*Drosophila melanogaster*; Sharon *et al*., 2010; Venu *et al*., 2014), locusts (*Schistocerca gregaria*; Dillon, Vennard, & Charnley, 2000, 2002), and termites (*Hodotermes mossambicus*; Minkley *et al*., 2006).

Bacteria facilitate the production of guaiacol, an insect aggregation pheromone that supports swarming behaviour, as observed in locusts (*Schistocerca gregaria*) (Dillon *et al*., 2000). Interestingly, more recent research suggests that swarming behaviour in the same locust species is also mediated by serotonin, which plays a role in the behavioural gregarisation that precedes swarming (Anstey *et al*., 2009). It remains unknown whether the bacteria-associated, guaiacol-mediated pathway underlying swarming is related to the serotonin-mediated pathway underlying gregarisation. Indirect evidence for such a connection derives from studies that investigate the effects of infection with the fungus *Paranosema locustae*, which both inhibits locust swarming behaviour *via* acidification of the hindgut and supresses seroton-inproducing bacteria (Shi *et al*., 2014). In this regard, investigating the possibility of a microbiome–guaiacol–serotonin system supporting gregarisation and swarming in locusts would be particularly interesting (Münger *et al*., 2018).

Microbially generated odours may also provide cues to recognise colony members. For instance, experimental alteration or disruption of the external microbiome of harvester ants (*Pogonomyrmex barbatus*; Dosmann *et al*., 2016) and the gut microbiome of lower termites (*Reticulitermes speratus*; Matsuura, 2001) interferes with nestmate recognition, leading to rejection of colony members. The microbiome may also contribute to insect reproductive behaviour. For example, it has been suggested that fruit flies *(Drosophila melanogaster*) show mating preferences for conspecifics with similar microbial compositions, a social cue attributed to *Lactobacillus plantarum* (Najarro *et al*., 2015; Sharon *et al*., 2010; but see Leftwich *et al*., 2017; see also Rosenberg *et al*., 2018).

### (4) Non-human mammals

Host-associated microbial populations in the gut or other dedicated scent-producing structures also contribute to mammalian social olfaction. Early observations of this phenomenon were made in the anal scent pouches of mongooses (*Herpestes auropunctatus*) (Gorman, Nedwell, & Smith, 1974; Gorman, 1976) and red foxes (*Vulpes vulpes*) (Albone *et al*. 1974; Albone & Perry, 1976). More recent work has examined bacterially mediated social olfaction in hyaenas.

The compounds in the scent gland secretions (paste) of spotted hyaenas contain bacterially derived odourants that are associated with the signalling of important social information (Theis *et al*., 2013), including host sex, immigration status in males, and pregnancy and lactation status in females (Theis *et al*., 2013). Furthermore, social groups of hyaenas are distinguishable on the basis of these bacterially generated odour profiles (Theis, Schmidt, & Holekamp, 2012; Theis *et al*., 2013). Other mammals in which microbial composition appears to correlate with social olfaction include badgers (*Meles meles*) (Sin *et al*., 2012; Noonan *et al*., 2019), meerkats (*Suricata suricatta*) (Leclaire, Nielsen, & Drea, 2014; Leclaire *et al*., 2017), and elephants (*Loxodonta africana* and *Elephas maximus*) (Goodwin *et al*., 2012).

Experimental efforts in rodents have begun elucidating the odourant molecules that are sensitive to the presence of microbes. For instance, the murine microbiome generates trimethylamine, which acts as an attractive olfactory cue. Antibiotic treatment reduces trimethylamine production, causing mice to become less sexually attractive to conspecifics (Li *et al*., 2013). Moreover, the urine of germ-free rats appears to lack biochemicals that are involved in individual identification based on odour discrimination (Singh *et al*., 1990), although this reduction in microbially derived odourants may not be sufficient to inhibit reproductive behaviour consistently (Nielsen *et al*., 2019).

### (5) Humans

At present, it is not known whether bacteria affect human social perception or social interaction via modulation of social olfaction. There is some evidence that the skin microbiome may contribute to human odour profiles, but overall, the association is weak at best and appears to be very sensitive to behaviours (e.g. bathing, deodorant use) and external factors (Xu *et al*., 2007). There is also evidence that the human skin microbiome produces compounds that act as attractants for mosquitoes, including the malaria mosquito *Anopheles gambiae sensu stricto* (Verhulst *et al*., 2010a, 2010b). These mosquitoes rely on odour profiles to target potential hosts, and both the composition of the skin microbiome and the compounds it produces can influence odour profiles and therefore the host’s attractiveness to mosquitoes (Verhulst et al., 2010a, 2010b, 2011). Overall, these results suggest that microbes affect human odour profiles. The finding that microbes contribute to human odour has implications for host health and infection, but it cannot necessarily be inferred that this microbial influence on odour profiles extends to human social perception or social interaction. While some studies do suggest a role for pheromones in human social interaction (Gildersleeve *et al*., 2012; Frumin *et al*., 2015), further research is required to determine the existence and effects of human pheromones (Wyatt, 2015).

## VIII. MICROBIAL ASSOCIATIONS WITH EMOTION AND SOCIAL BEHAVIOUR IN HUMANS

### (1) Psychobiotic studies

Compared to the relatively clearer psychobiotic effects on rodent behaviour, human research has not found consistent psychological benefits of probiotic consumption (Kelly *et al*., 2017). However, there are some important parallels with rodent findings. For example, consumption of psychobiotics lowers cortisol levels (Messaoudi *et al.,* 2011; Schmidt et al., 2015; Allen *et al*., 2016) and is accompanied by self-reported reductions in negative mood (Messaoudi *et al*., 2011; Steenbergen *et al*., 2015). In a recent double-blind, randomised psychobiotic administration experiment, *Bifidobacterium longum* 1714 consumed over a four-week period was found to affect brain activity associated with the psychological stress induced by social exclusion, as measured by magnetoencephalography (Wang *et al*., 2019). Relative to participants treated with a placebo, those treated with the psychobiotic displayed increased resting-state *θ* power in the frontal and cingulate cortices, and reduced *β*2 power in the hippocampus, the fusiform gyrus, the temporal cortex, and the cerebellum (Wang *et al*., 2019). In response to the social task, participants treated with the psychobiotic (relative to the placebo) also showed increased power in the *θ* and *α* bands in several brain regions, including the inferior, medial, and superior frontal cortices, the anterior and middle cingulate cortices, and the supramarginal gyrus (Wang *et al*., 2019). While these results cannot necessarily be linked to a particular psychological state or experience, they do suggest that psychobiotics may be capable of modulating brain activity both at rest and in response to social experiences. However, as with most other human studies, the sample sizes were relatively small.

We have hypothesised that one mechanism underlying some of the psychological effects of psychobiotics may be a generalised decrease in social–emotional reactivity (Sarkar *et al*., 2018). For example, consuming probiotics has been found to reduce activity in a brain network associated with processing emotional information in response to facial stimuli (including the amygdala) (Tillisch *et al*., 2013), and another study showed that probiotic consumption reduced psychological reactivity to sadness (Steenbergen *et al*., 2015). There is also evidence that prebiotics can reduce waking cortisol levels and emotional attention to negative stimuli (Schmidt *et al*., 2015).

### (2) Microbiome–depression associations

Disorders of emotion, such as depression, often exert profound effects on normal human social behaviour, and are characterised by a loss of interest in pleasurable activities (including social interactions) as well as social withdrawal and isolation. There is much interest in characterising emotional disorders in terms of consistent bacterial signatures. For instance, depression has recently been associated with changes in the relative abundance of numerous bacterial taxa. These include increases in the Firmicutes phylum, decreases in the Bacteroides phylum, and increases in the genera *Prevotella, Klebsiella, Streptococcus* and *Clostridium* (Lin *et al*., 2017). Others have found increases in the Enterobacteriaceae family and the *Alistipes* genus and decreases in the *Faecalibacterium* genus in depressed individuals relative to healthy controls (Jiang *et al*., 2015), or order-level increases in Bacteriodales and family-level increases in Lachnospiraceae (Naseribafrouei *et al*., 2014). Some studies have found increases in Actinobacteria and Proteobacteria in depressed individuals (Jiang *et al*., 2015; Zheng *et al*., 2016).

Comparing studies reveals some contrasting results, with studies reporting evidence of depressed individuals showing both higher (Naseribafrouei *et al*., 2014; Jiang *et al*., 2015) and lower (Zheng *et al*., 2016) levels of Bacteroidetes. In some cases, the abundance of particular bacterial taxa correlates with the severity of depression. For instance, a negative association was found between the relative abundance of *Faecalibacterium* spp. and the severity of depressive symptoms (Jiang et al., 2015). Following up on the need for larger studies, a recent metagenomic survey in two large European samples reported evidence that depression is associated with reduced levels of *Coprococcus* spp. and *Dialister* spp., even after controlling for antidepressant treatment (Valles-Colomer *et al*., 2019).

The psychological implications of variation in particular bacterial communities for emotional disorders remain rather unclear. At present, it is largely unknown how different bacterial communities might contribute to depression, although perhaps some cautious inferences can be drawn from specific functions of bacteria that have been examined in other contexts. For example, *Alistipes* spp. may be linked to increased inflammation (Naseribafrouei *et al*., 2014), which is often a prominent physiological marker of depression (Miller, Maletic, & Raison, 2009). Other recent research has found that several human-associated bacterial genera produce GABA or use it as a nutrient (Strandwitz *et al*., 2019). For instance, growth of the bacterial isolate KLE1738 appears to depend on GABA as a nutrient, which is produced by members of the *Bacteroides* genus under pH conditions similar to the human gut (Strandwitz *et al*., 2019). Moreover, in a small sample of clinically depressed individuals, the relative abundance of the genus *Bacteroides* was negatively correlated with brain signatures of depression (Strandwitz *et al.*, 2019). Specifically, reduced *Bacteroides* abundance was linked to stronger functional connectivity between the left dorsolateral prefrontal cortex and the default mode network (Strandwitz *et al*., 2019), and such increased functional connectivity has previously been associated with depression.

### (3) Infancy and early development

There is strong interest in the changes in microbiome composition during infancy and early development. The mammalian neonate’s microbiome is shaped by numerous environmental influences, one of the first of which is breast-milk (Allen-Blevins, Sela, & Hinde, 2015). Breastmilk provides, for instance, an important supply of prebiotic glycans (human milk oligosaccharides) to the infant gut (Charbonneau *et al*., 2016). In infants and young children, microbial composition has been shown to correlate with temperament and emotional regulation (Christian *et al*., 2015; Aatsinki *et al.,* 2019), as well as cognitive development and linguistic skill (Carlson *et al*., 2018), which are beneficial for social interaction.

One particularly important area in microbiome research that may be relevant to the social–emotional development of humans is the effect of early antibiotic exposure, given the rising prevalence of antibiotic use (Blaser, 2016; Sonnenburg & Sonnenburg, 2019), especially among young children (Cox & Blaser, 2015). For example, murine studies have found that exposure to low doses of antibiotics during infancy can permanently alter the host’s gut microbiome and endocrine physiology (Cho *et al*., 2012). In addition, antibiotic treatment in young mice has been found to reduce the expression of neuroreceptors implicated in social and emotional behaviour, namely *μ*-opioid, oxytocin, and vasopressin receptors (K.V.-A. Johnson & P.W.J. Burnet, in preparation). In humans, antibiotic administration in early life has been associated with greater incidence of depressive symptoms in later childhood (Slykerman *et al*., 2015). Similarly, antibiotics administered in early life were associated with negative outcomes on measures of cognitive function even at 11 years of age, after adjusting for other variables such as probiotic exposure and breastfeeding (Slykerman *et al*., 2019). These studies add inductive support to the hypothesis that a healthy microbiome in early life is important for typical social–emotional development in humans, and that antibiotics may disrupt this development. However, these investigations (Slykerman *et al*., 2015, 2019) did not directly examine antibiotic effects on microbial composition. Therefore, while variations in subsequent psychological outcomes are certainly consistent with the possibility of antibiotic-induced microbial disturbances, they may also arise from modulation of non-microbial targets. For example, they may be associated with the many off-target effects of antibiotics, such as those described in Section II. Alternatively, because antibiotics are administered in response to infection, the observed increase in childhood depression may be attributable to elevated inflammation caused by the infection for which antibiotics were used in the first instance. This is plausible given that inflammation and depression are often robustly associated (Dowlati *et al*., 2010), and that childhood inflammation can predict future depression in young adults, even several years later (Khandaker *et al*., 2014). Therefore, the finding that antibiotic exposure predicted depressive symptoms may simply indicate that the infants suffered from illness (and inflammation), rather than the depressive symptoms occurring in response to antibiotic-induced microbial perturbations. Future studies of this type should also incorporate analyses of the microbiome following antibiotic administration, which will provide a clearer understanding of the relationship between the microbial and psychological changes associated with antibiotic use.

The infant microbiome may also be sensitive to prenatal stress during pregnancy. For example, in the first 110 postnatal days, higher levels of maternal prenatal stress were found to be associated with shifts in infant microbial composition that, in turn, were associated with greater levels of inflammation and poorer health outcomes (Zijlmans *et al*., 2015). These human results appear similar to murine findings. In particular, it is important to keep in mind that maternal prenatal stress alters the vaginal microbiome (Jašarević, Morrison, & Bale, 2016). The vaginal microbiome is important in this context because it is assumed to be the first microbial exposure for mammalian infants, with vaginal microbes colonising the infant gut microbiome during parturition (Dominguez-Bello et al., 2010; Mueller *et al*., 2015; Sprockett *et al*., 2018). A stress-associated vaginal microbiome in female mice can be transmitted to the infant during birth, which in turn can impact the developing infant’s health, metabolism, and stress response (Jašarević *et al*., 2015, 2018). While the actual vertical transmission of stress-associated microbes has not yet been observed in humans, researchers have detected stress-associated microbial changes in the maternal human gut during pregnancy (Hechler *et al*., 2019). In addition, it has been shown that prenatal stress in pregnant monkeys alters the microbial composition of the infant gut (Bailey, Lubach, & Coe, 2004). Thus, it is plausible that vertical transmission of stress-associated microbes to the infant during vaginal births may occur in humans as well, with neurodevelopmental implications for the infant. Conclusive evidence for this phenomenon would require longitudinal studies of both maternal and infant microbiomes over time, alongside tracking of maternal and infant stress.

### (4) Potential prenatal microbial exposures

In humans (and mammals more generally) the conventional view is that the womb is a germ-free environment. For mammals, the earliest colonisation event is believed to occur during parturition. The mother’s vagina serves as the infant’s first source of microbes, and it is assumed that there is no microbial exposure *in utero*.

Some researchers have questioned this ‘sterile womb’ hypothesis, suggesting that microbial exposures also occur *in utero* (Funkhouser & Bordenstein, 2013). Researchers have already identified a microbe → maternal physiology → foetus pathway (i.e. indirect microbe–foetus contact, as studied, for instance, by Kim *et al*., 2017). However, the possibility of prenatal exposures dramatically changes the nature of microbial influence on the foetus, as it would imply a mother → microbe → foetus pathway (i.e. direct microbe–foetus contact), with the possibility of prenatal microbial colonisation. To this extent, researchers found what appeared to be a unique placental microbiome (Aagaard *et al*., 2014; Antony *et al*., 2015), which suggests that microbial populations might be able to reach and colonise the foetus. Others have taken this possibility further by proposing the existence of an amniotic microbiome and a foetal microbiome (Collado *et al*., 2016; Martinez II *et al*., 2018). Such prenatal microbial exposures, if they existed, could profoundly alter the current understanding of mammalian developmental biology.

However, these intriguing possibilities are challenged by findings that the presence of microbes may instead result from methodological artefacts such as reagent contamination (Lauder *et al*., 2016; Perez-Muñoz *et al*., 2017; Leon *et al*.,2018; Lim, Rodriguez, & Holtz, 2018; de Goffau *et al*., 2019; Theis *et al*., 2019). Moreover, it is also the case that some potentially pathogenic microbes, such as Streptococcus agalactiae, may indeed be capable of infecting the placenta, with implications for neonatal health (de Goffau *et al*., 2019). However, the presence of potential pathogens in the placenta cannot be interpreted as evidence that there is also an intrinsic or typical placental microbiome (comprising mutualists, commensals, and pathobionts). Rather, *Streptococcus agalactiae* appears to occur in a minority of cases, and its presence is considered atypical and infectious (de Goffau *et al*., 2019).

Bushman (2019) provides a useful historical overview of the issues regarding the placental microbiome. At present, the existence of placental, amniotic, or foetal microbiomes, although intriguing, remains controversial and requires rigorous confirmatory evidence.

### (5) Social behaviour and autism

Any effect of the microbiome on human sociality is expected to occur through the mechanisms inferred from studies using mammalian models. In practice, however, testing this association will be extremely challenging, not least because of a lack of adequate animal models of human social development and the necessary ethical limitations of experimentation in humans (although primate models of the kind described in Section III above may provide further insight). There are few observations of microbial effects on human social behaviour, though researchers are particularly interested in the microbiome–autism link, which entails analyses of social behaviour by definition.

At the observational level, a number of studies have attempted to differentiate between autistic and neurotypical children on the basis of microbiome composition. For example, surveys of autistic individuals have found decreased levels of *Coprococcus, Prevotella*, and Veillonellaceae compared to healthy controls (Kang *et al*., 2013), and elevations in *Clostridium* (Finegold *et al*., 2002; Song, Liu, & Finegold, 2004; Parracho *et al*., 2005) and *Sutterella* (Williams *et al*., 2011; Wang *et al*., 2013). At the same time, there is also considerable variation and discrepancy in identifying bacterial markers of autism. For example, the ratio of the Firmicutes to Bacteroidetes phyla in autistic compared to non-autistic children has been found to be elevated, reduced, or unchanged in different studies (Finegold *et al*., 2010; Williams *et al*., 2011; Kang *et al*., 2013; Son *et al*., 2015; Forsythe *et al*., 2016). A recent systematic review of 16 studies did find cross-study evidence of some consistent microbial differences in autistic individuals compared to neurotypical controls, including increased *Bacteroides, Clostridium, Desulfovibrio, Lactobacillus, and Proteobacter,* and decreased *Bifidobacterium, Blautia, Dialister, Prevotella, Veillonella*, and *Turicibacter* (Liu *et al.,* 2019). At present, it is unclear how specific bacterial populations might contribute to the pathophysiology of autism, but researchers are attempting to characterise the physiological roles that these bacteria play (e.g. modulation of inflammation and metabolism). This may then help researchers infer how altered relative abundances in different bacterial populations may be used to characterise at least some of the features of autism. In addition, a recent study employing multiple regression analyses found that certain bacterial genera previously associated with autism are also significantly related to individual differences in sociability in neurotypical adults, and in the same direction as typically found in autistic individuals (Johnson, 2020). It has therefore been suggested that the gut microbiome may contribute to variation in social behaviour in the general population, as well as in autism (Johnson, 2020).

The possibility that autism may be associated with distinct microbial profiles in humans has led to a great deal of interest in modifying the microbiome in an attempt to target autism-associated behaviours. These approaches have yielded varying rates of success. For instance, one probiotic administration study that implemented a double-blind, crossover design failed to detect changes in behaviour in autistic participants, but did observe some differences in microbial composition (Parracho *et al*., 2010). In another intervention study, researchers found that antibiotic treatment with vancomycin over an eight-week period mitigated behavioural phenotypes in a small sample of autistic children (Sandler *et al*., 2000). However, these benefits were transient, and were mostly absent within just 2 weeks following vancomycin treatment, and were also absent at long-term follow-ups (Sandler *et al*., 2000). Furthermore, though some antibiotics may provide short-term benefits (e.g. Sandler et al., 2000), it is likely unfeasible to engage in chronic antibiotic treatment for autism, as there is presently no way of controlling the detrimental effects on the microbiome, as well as the inevitable development of antibiotic resistance that prolonged exposure would induce.

Recently, researchers have adopted a more direct approach to modifying the microbiome: an open-label investigation in a sample of 18 autistic participants investigated the efficacy of faecal transplants in treating gastrointestinal and behavioural symptoms (Kang *et al*., 2017). In order to deplete as many gut bacteria as possible, participants first underwent broad-spectrum antibiotic treatment using vancomycin for 2 weeks, and were then given a bowel cleanse to remove any remaining bacteria and vancomycin. They were also given an acid suppressant to reduce stomach acidity, which would facilitate survival of orally administered microbes. Following this, participants received faecal transplants from neurotypical donors over several weeks (first at a high initial dose that was delivered orally or rectally, followed by lower maintenance doses administered orally). More precisely, rather than transferring pure faecal matter, donor faeces were used to generate a standardised human gut microbiome, containing over 99% bacteria (Hamilton *et al*., 2012). At the end of the treatment, participants showed substantial improvement in both gastrointestinal symptoms (e.g. diarrhoea and indigestion), and social deficits and other behavioural features (e.g. repetitive behaviours). Participants were also reported to have gained 1.4 years in developmental age on measures of adaptive behaviours (e.g. communication and living skills). These improvements were apparent 8 weeks following the cessation of treatment. In addition, the researchers detected elevations in *Bifidobacterium*, Desulfovibrio, and Prevotella which also remained 8 weeks after treatment (Kang *et al*., 2017). Even more striking were the results of follow-up assessments conducted on these participants 2 years following the completion of the microbial transplant: most of the gastrointestinal and behavioural improvements had persisted through the intervening period, and several of the autism-related symptoms had improved even further (Kang *et al*., 2019). Moreover, the elevations in *Bifidobacterium* and *Prevotella* remained (Kang *et al*., 2019). By showing that some of the microbial changes were preserved in the recipient gut even 2 years later, these results also extend earlier findings that transferred faecal microbes can survive in the recipient for at least a few months (Li *et al*., 2016).

These results suggest that the human microbiome may serve as a therapeutic target in the treatment of autism. However, placebo-controlled, double-blind, randomised trials with larger samples are required to better understand the therapeutic potential of microbial transfers. In an earlier article (Sarkar *et al*., 2018), we suggested that one reason that microbiome transplants may yield greater therapeutic efficacy for autism compared to psychobiotic or antibiotic routes is the difference in scale: the number of microbes that can be introduced into a new host *via* faecal transfers is many orders of magnitude greater than probiotic consumption. Typical probiotic doses can only introduce a comparatively small number of microbes into the gut, and, as discussed earlier, these are often unsuccessful in colonising the new host. Furthermore, in comparison to probiotic treatment, which typically involves the administration of only one or a few bacterial strains, a faecal transfer can introduce an entire bacterial community into the recipient’s gut.

Of course, while these results (Kang *et al*., 2017, 2019) are promising, the small initial sample size (*N* = 18), the open-label nature of the design, and the lack of a control group all pose substantial challenges to the generalisability and applicability of these results. For example, a small sample size combined with a high degree of variance can often result in an overestimation of the true effect size (Gelman & Carlin, 2014). Thus, it may be that even if this approach yields therapeutic benefits for autistic individuals, the average improvement may be smaller than that observed in this sample.

### (6) Statistical power, replication, and causal evidence

As noted elsewhere (Forsythe *et al*., 2016; Sarkar *et al*., 2018), human research on the link between the microbiome and psychological processes is fraught with noise arising from variations in genetics, sex, age, diet, past and present environmental exposures, and use of medicines, all of which can be strictly controlled in laboratory-based rodent studies. While some of the stress- and emotion-related findings in humans resemble rodent findings in several respects, they have much lower statistical power. Moreover, some experiments and meta-analyses have not found consistent psychological effects of probiotic consumption (Romijn & Rucklidge, 2015; Kelly *et al*., 2017; Romijn *et al.*, 2017). Overall, while many of these findings are promising, they must also be viewed as preliminary, and highlight the need to examine the psychological and social effects of intrinsic microbial variation and exogenous microbial manipulation in larger and more diverse samples.

In general, there is limited evidence that the results obtained in one study will be reliably replicated in subsequent studies. This is especially true for the neuroimaging research we have described here. Given the relatively small sample sizes in these reports, alongside the known prevalence of very low statistical power in cognitive neuroscience and brain-imaging research (Button *et al*., 2013; Szucs & Ioannidis, 2017), it may be that many of the most intriguing microbiome–brain associations in humans are false positives. Thus, until replications have been conducted, it would be prudent to be at most cautiously optimistic about these associations.

It should also be kept in mind that these findings are instances of correlation (in many cases with low statistical power to detect effects). While causal speculation is of course permissible for the generation of hypotheses and design of future studies (particularly in light of evidence from animal research), most of the human findings do not provide any direct evidence of causation.

## IX. CONNECTING PHYSIOLOGY TO SOCIAL BEHAVIOUR

### (1) Two types of investigations

Investigations of microbiome-associated changes in the host that are relevant to host social behaviour can broadly be placed in one of two categories. The first category consists of studies that analyse the effect of the microbiome (e.g. *via* germ-free animals or antibiotic administration) on concentrations of molecules implicated in social behaviour, or the structure and function of relevant brain regions. However, social behaviour itself is frequently not measured in these studies. For instance, the pronounced influence of the microbiome on endogenous testosterone concentrations (Markle *et al*., 2013) was discovered in the context of autoimmunity, and the motivation for the research more broadly was the immunosuppressive – not the social – effect of testosterone. The second category comprises studies that investigate microbial effects on social behaviour, and also examine microbial effects on host physiology in parallel. However, in most cases, the relationships between the behavioural and physiological effects uncovered by these studies are correlational. Thus, it is often rather difficult to interpret the direction of causality, or which biological changes mediate the relationship between the microbiome and assays of host social behaviour.

### (2) Connecting the microbiome to social behaviour

There is limited research that conclusively identifies a biological mediator of the relationship between the microbiome and host sociality, although of course such mediators must exist. While it is likely that the microbiome–sociality relationship is mediated, at least in part, by changes in the anatomy and function of regions in the social brain, or in the biosynthesis and bioavailability of social signalling molecules, there are few studies that have identified such underlying pathways from changes in the microbiome to changes at the behavioural level.

Consider the involvement of the microbiome in autism. In terms of the neurological basis of the microbiome–autism connection, our current knowledge is based on adjacent links in a chain. One link, supplied by microbiology and neuroscience, is the finding that the microbiome influences amygdalar structure and function (Luczynski *et al*., 2016; Hoban *et al*., 2018). The second link, from cognitive neuroscience and biological psychiatry, is the finding that variations in amygdala structure and function may be involved in autism (Baron-Cohen *et al*., 1999, 2000). However, these findings cannot automatically be connected to infer that the amygdala plays a role in the microbiome’s interactions with autism in humans (or even in mice, for that matter). Indeed, microbiome-associated changes in the amygdala may only share minimal overlap with autism-associated changes in the amygdala. Therefore, at best, the current set of findings permits the possibility that a microbiome → amygdala → autism connection *may* exist and could be subject to future investigation.

Similarly, consider the example of testosterone. One link in the chain is that the microbiome affects testosterone, and the adjacent link, supplied by behavioural endocrinology, is that testosterone affects animal social behaviour. In rodents, this would likely manifest as aggression. But there is as yet no report of a microbiome → testosterone → aggression connection in rodents. Until evidence of such a link is generated, we cannot know whether microbial effects on testosterone actually influence social behaviour. Even when the microbiome does influence testosterone bioavailability, the hormone may not necessarily affect behaviour, since there are many different physiological actions of testosterone, some of which may have no significant behavioural correlates. Furthermore, it is important to keep in mind that all of these molecules (neurotransmitters, steroids, and neuropeptides) perform numerous physiological functions for the host, and variations in their bioavailability cannot be assumed to exert psychological effects on the host.

### (3) Linking microbes to social behaviour *via* a biological mediator

To further our understanding of the microbiome–sociality connection, explicit investigations are required into how the physiological changes induced by the microbiome influence behaviour. The investigation of the microbiome–autism connection by Kim *et al*. (2017) is an example in this regard. The researchers found that the presence of segmented filamentous bacteria in the maternal gut is necessary for maternal immune activation to trigger autistic-like traits in the offspring. These findings reveal mechanistic connections between the maternal microbiome and offspring social behaviour that are mediated by the action of interleukin-17a secreted by maternal T_H_17 cells.

Another example is the finding that *Lactobacillus reuteri* only ameliorates social deficits in mice with functioning oxytocin systems, as conditional deletion of oxytocin receptors in neurons in the ventral tegmental area prevented Lactobacillus reuteri treatment from rescuing social impairments (Sgritta et al., 2019). Therefore, this experiment provides valuable evidence of a bacterium → oxytocin → social behaviour relationship. This type of investigation helps connect bacteria to behaviour *via* a likely physiological mediator (oxytocin), thereby providing evidence of a causal pathway. Of course, the causal pathway itself will be substantially more complex than this, involving a number of other signalling molecules and components (e.g. the vagus nerve), but at the very least, we can begin to consider how a social signalling molecule plays a role in the microbiome–sociality relationship.

### (4) Other signalling molecules

In this article, we have focussed mainly on a specific set of molecules that have well-documented effects on social behaviour. However, the microbiome regulates a wide range of other molecules and some of these may also influence animal social behaviour. For instance, researchers have recently found evidence suggesting that the proinflammatory cytokine interferon-γ may play a role in social behaviour across the animal kingdom (Filiano *et al*., 2016). They hypothesise that this link between interferon-γ and social behaviour may have arisen over evolutionary time during the transition to sociality since group living may have favoured a stronger immune response to protect organisms from pathogens transmitted by conspecifics (Filiano *et al*., 2016). Some probiotics are able to alter the concentrations of interferon-γ, as well as other proinflammatory cytokines (Desbonnet et al., 2008; Donato *et al*., 2010; Rodrigues et al., 2012). While it is presently unknown whether microbiome-related variations in cytokines can affect social behaviour, the discovery of central lymphatic vessels that could deliver immune molecules to the brain suggests that the connection between the immune system and the brain is more direct that previously thought (Louveau *et al*., 2015). Given the relationship between the microbiome and the immune system (Round & Mazmanian, 2009; Fung *et al*., 2017) as well as between the immune system and social behaviour (Eisenberger *et al*., 2017), it is at least conceivable that some of the microbiome–sociality connections may be mediated by immune molecules.

## X. UNDERSTANDING THE ORDER AND NATURE OF MICROBIAL EFFECTS ON HOST SOCIALITY

**Fig. 6.**
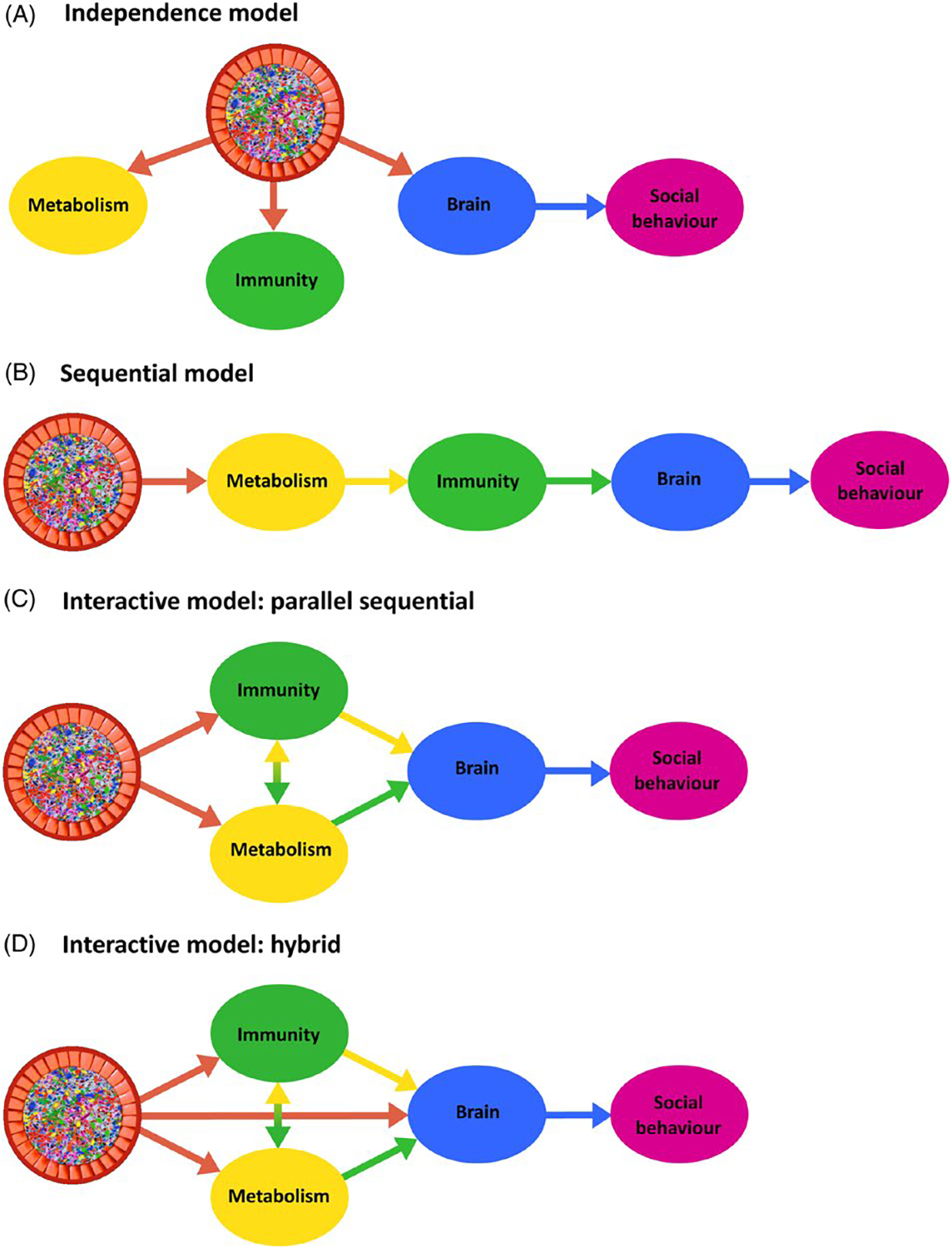
Models of the order of microbial effects on host social behaviour. Germ-free models, as well as studies involving antibiotic administration or disruptions to maternal physiology during pregnancy, have revealed the important contributions of the microbiome to host development in rodents. However, because many of the specific mechanisms by which microbes influence host physiological development are unknown, it is currently not possible to establish the order of these effects, and how they in turn affect social behaviour. This gives rise to several hypotheses about how these processes occur in relation to one another. (A) The microbiome influences the immune system, metabolism, and the brain independently, with microbial effects on the immune system and metabolism having little or no effect on the brain and social behaviour. (B) A sequential model in which a particular developmental sequence leads to the microbiome ultimately affecting host social behaviour. The microbiome influences the development of a single function, which in turn influences a second function, and so on. A and B are unlikely to occur in reality, but may represent useful null models for the generation of predictions and comparisons. (C) An interactive model which assumes that the microbiome makes core, parallel contributions to host metabolism and immunity that interact with one another, and which in turn influence brain development and function, contributing to the host’s social behaviour (in this case, effects on the brain follow sequentially from changes in host metabolism and immunity). (D) An interactive hybrid model. Apart from microbial effects on the brain via immunological and metabolic influences, brain development may also be affected by the microbiome more directly, perhaps via modulation of the vagus nerve or enteric nervous system. These models are highly simplified, and represent merely four possibilities. Many other combinations are of course possible, and may involve other physiological systems including the endocrine system and the olfactory system. Although this diagram only shows microbial effects on host physiological functions, the relationships are bidirectional, with the host’s metabolism, immunity, brain, and social behaviour exerting effects on the microbiome as well.

Since the specific mechanisms by which the microbiome influences host physiology remain poorly understood, an important and currently unresolved question is the order of microbial effects on host social development and behaviour (see Fig. 6). Germ-free status has been linked to many physiological impairments, supporting the claim that microbes are essential for normal development. Since animal life evolved in the presence of microbes, it can be expected that a total absence of microbes would alter normal physiology (McFall-Ngai *et al*., 2013). Indeed, it is difficult to overstate the extent of dysfunction in germ-free animals, including supressed angiogenesis (Stappenbeck, Hooper, & Gordon, 2002), abnormal stress reactions (Sudo et al., 2004), abnormal immune development (Olszak *et al*., 2012), abnormal development of the enteric nervous system (McVey Neufeld *et al*., 2013), excessive permeability of the blood–brain barrier (Braniste *et al*., 2014), and abnormal brain development (Diaz Heijtz *et al.*, 2011; Hoban *et al*., 2016, 2017; Luczynski *et al*., 2016).

Despite the many deficits of germ-free animals, it is not known how the numerous microbial effects on host physiological, psychological, and social development are connected to one another. Are microbial contributions to social behaviour purely reflective of what may be considered their core contributions to metabolic and immunological development? Perhaps the changes in one function are in fact caused by changes in another function, and may in turn trigger further changes. Or is it that microbial effects on neurotransmitters, brain circuitry, the endocrine system, and the olfactory system, all of which play key roles in sociality, arise independently of microbial regulation of host metabolism and immunity? This latter proposition is unlikely, but may have some utility as a point of comparison for more probable models. Researchers face significant challenges – and opportunities – in establishing the causal order of bacterial contributions to host physiological development, and this in turn will allow for more precise examination of microbial contributions to social behaviour. For example, to investigate the overall influence of the microbiome on social behaviour, researchers could longitudinally administer a battery of physiological and social tests to germ-free mice colonised at different ages with microbiomes from healthy and socially atypical conspecifics. Researchers could also administer the same physiological and social tests to conventional mice treated with broad-spectrum antibiotics and compare the results to the control group with matched ages. Then the microbiomes of a subset of these antibiotic-treated mice might be ‘restored’ *via* transplants from socially normal *versus* socially atypical conspecifics to gain insight into the extent to which physiology and social behaviour are transmissible *via* the microbiome, given an initially healthy phenotype.

## XI. CONCLUSIONS

1. The microbiome affects animal social behaviour, but the specific biological pathways that mediate these associations are yet to be fully elucidated.
2. There is evidence that changes in the gut microbiome can lead to changes in the structure and function of the social brain, influence various neurochemicals, and alter genetic and epigenetic processes.
3. Microbiome–sociality associations include relationships between the microbiome and social stress, social interaction, and autistic phenotypes.
4. The microbiome affects the development and function of regions of the social brain, including the amygdala, prefrontal cortex, hippocampus, and hypothalamus.
5. The microbiome influences the concentrations and signalling properties of a variety of molecules that play an important role in social behaviour (i.e., social signalling molecules), including glucocorticoids such as cortisol and corticosterone, sex steroids such as testosterone, oestradiol, and progesterone, neuropeptides such as oxytocin and vasopressin, and monoamines such as serotonin and dopamine.
6. The microbiome is associated with changes in RNA biology and gene expression that may relate to host social behaviour.
7. The microbiome generates a range of olfactory signalling molecules (odourants) that can bind to olfactory receptors distributed throughout the host’s body. This microbiome–olfaction–behaviour pathway may play a more important role in host sociality than is currently recognised. Microbially produced signalling molecules also influence host social interactions with conspecifics in both insects and mammals *via* the regulation of host odour profiles and scent-based markings (e.g. urine, faeces, and paste).
8. Observational and psychobiotic studies in humans suggest that the microbiome is involved in human emotional processes as well. Several psychobiotics appear to be beneficial in reducing negative emotions in humans. Researchers have also detected correlations between microbial composition and depression, although the functional role of bacteria in depression remains largely unknown. The microbiome in early infancy may also play a role in the development of social and emotional traits, and may even influence individual susceptibility to developing autism. At the same time, human microbiome research is typically underpowered and the data are characterised by a great deal of variation. Perhaps because of this, studies investigating the relationship between the human gut microbiome and psychology sometimes report inconsistent findings.
9. There is limited direct evidence connecting the microbiome to a specific social behaviour *via* a physiological mediator. As such, designing experiments to examine these links should be prioritised in future research.
10. It will be important to understand the order of microbial effects on host sociality, since the microbiome affects multiple aspects of physiology, including the nervous system, metabolism, and immunity.
